# Alternative transcript splicing regulates UDP-glucosyltransferase-catalyzed detoxification of DIMBOA in the fall armyworm (*Spodoptera frugiperda*)

**DOI:** 10.1038/s41598-022-14551-w

**Published:** 2022-06-20

**Authors:** Bhawana Israni, Katrin Luck, Samantha C. W. Römhild, Bettina Raguschke, Natalie Wielsch, Yvonne Hupfer, Michael Reichelt, Aleš Svatoš, Jonathan Gershenzon, Daniel Giddings Vassão

**Affiliations:** Max Planck Institute for Chemical Ecology, Jena, Germany

**Keywords:** Biochemistry, Molecular biology, Ecology, Molecular ecology

## Abstract

Herbivorous insects often possess the ability to detoxify chemical defenses from their host plants. The fall armyworm (*Spodoptera frugiperda*), which feeds principally on maize, detoxifies the maize benzoxazinoid 2,4-dihydroxy-7-methoxy-1,4-benzoxazin-3-one (DIMBOA) by stereoselective re-glucosylation using a UDP-glucosyltransferase, SfUGT33F28. SfUGT33F28 activity is induced by feeding on a DIMBOA-containing diet, but how this induction is regulated is unknown. In the present work, we describe the alternative splicing of the *SfUGT33F28* transcript. Variant transcripts are differentially expressed in response to DIMBOA, and this transcriptional response is mediated by an insect aryl hydrocarbon receptor. These variants have large deletions leading to the production of truncated proteins that have no intrinsic UGT activity with DIMBOA but interact with the full-length enzyme to raise or lower its activity. Therefore, the formation of *SfUGT33F28* splice variants induces DIMBOA-conjugating UGT activity when DIMBOA is present in the insect diet and represses activity in the absence of this plant defense compound.

## Introduction

The fall armyworm (FAW, *Spodoptera frugiperda* J.E. Smith, Lepidoptera: Noctuidae) is a generalist-feeding insect pest that is found on more than 350 plant species, particularly on grass crops such as maize, rice, and sugarcane. The worldwide spread of the FAW from its native Latin America to Sub-Saharan Africa in 2016^1^, to Asia in 2018^2,3^ and more recently to Australia in 2020^4^ is unprecedented in its extent and speed. This insect now causes an annual loss of 8.3 to 20.6 million tons of maize in Africa alone, a value of US$ 2 to 6 billion^5^.

The ability of FAW and other insect herbivores to thrive on their host plants depends in part on detoxification processes, for example involving the conjugation of plant defense compounds to sugars mediated by uridine diphosphate-glycosyltransferases (UGTs). The products of UGT conjugation generally have increased solubility in water and greater chemical stability, and thus lower toxicity. Plant defenses detoxified by UGTs include terpenoids^6^, phenolics^7^ and various nitrogen-containing compounds^8,9^. UGTs are also principal detoxification enzymes in other organisms, such as mammals, and have additional functions in metabolism^10,11^. In insects, UGTs play roles in odorant clearance^12^, cuticle formation^13^, and pigmentation^14^, in addition to detoxification.

The FAW employs a UGT enzyme to detoxify the toxic benzoxazinoid aglucone DIMBOA, one of the major anti-herbivore defense compounds of maize. SfUGT33F28 catalyzes the re-glucosylation of DIMBOA, which is stored as a glucoside in maize leaves, but released as a toxic aglycone upon herbivory as a result of plant glucosidase activity^15,16^. Benzoxazinoid aglucones are usually unstable and can react with biological nucleophiles (e.g., protein thiol and amine groups) leading to enzyme inhibition^17^, or spontaneously degrade to form toxic benzoxazolinones. The stereo-selective insect-mediated conjugation, however, circumvents this by generation of (*2S*)-DIMBOA-Glc, an epimer of the plant-produced (*2R*)-DIMBOA-Glc, which is unsuitable as a substrate for the plant glucosidases that are still active in the insect gut^16^ and therefore not subject to activation. We further discovered that different FAW host races, which exhibit varying preferences for DIMBOA-containing maize, have different levels of DIMBOA glucosylation activity^15^. Thus, we investigated the regulation of UGT-catalyzed DIMBOA glucosylation in the FAW focusing on the transcription of *UGT33F28*. Expression of this gene has been previously shown to be induced upon exposure to DIMBOA in the diet^23^.

Prior studies on human UGTs indicate that transcription of this gene family can be regulated by alternative splicing. Human UGTs are broadly classified into two gene families, UGT1 and UGT2^18^. The UGT1A gene cluster consists of 13 promoters and unique first exons along with four common exons that together encode nine functional UGT1A enzymes via alternative promoter and exon usage^19^. For UGT2, ten enzymes are produced from nine genes through differential splicing utilizing an alternative first or second exon^20^. Alternative splicing therefore serves as a prominent means for generating a diversity of UGT isoforms, and interestingly, the expression of these variants can be triggered by xenobiotics^21,22^. Furthermore, isoforms interact physically leading to an expansion of the repertoire of detoxification reactions mediated by UGTs^23,24^. Hetero-dimerization can involve both N- and C-terminal domains^25^ and introduce conformational changes in the substrate binding site, influence co-factor binding and even alter the regio-selectivity of specific UGTs^23,24,26,27^.

In insects, relatively little is known about the transcriptional diversity of the UGT gene family. Expression of the *Helicoverpa armigera UGT33B2* gene, for instance, produces two different transcript variants, with one showing an internal deletion of 220 bp^28^. Such deletions can potentially shift the translation frame and cause the introduction of a premature termination codon. In *Bombyx mori*, *UGT46A1* and *UGT46A2* result from alternative exon usage, while unspliced introns have been identified in three *H. armigera* UGTs^28^. Splice site variation has also been reported in *Drosophila melanogaster*^29^, often resulting from exon skipping events leading to frameshifts. However, whether such transcript variants lead to proteins that retain catalytic function has not often been tested.

During our investigations of *S. frugiperda UGT33F28*, we detected a small number of alternative transcripts with truncations in the coding sequence. Here we characterize two *UGT33F28* transcript variants produced by *S. frugiperda* larvae. While the canonical full-length *SfUGT33F28* transcript is represented by four exons, the transcript variants are distinguished by deletions in exon 1 (transcript variant 1, tv1) and exons 2 and 3 (transcript variant 2, tv2), respectively. We show that the expression of these variants is regulated by dietary xenobiotics including DIMBOA, with participation of the aryl hydrocarbon receptor complex, a well-studied regulator of cellular responses to various xenobiotics. Furthermore, functional characterization of the variant proteins in vitro in insect cells demonstrated that they have no DIMBOA-UGT activity of their own, but they retain the ability to interact with the full-length SfUGT33F28 protein to increase or decrease its activity towards DIMBOA. Alternative splicing thus modifies the protein architecture and changes the rate of catalysis, allowing a fine-tuning of the insect UGT activity.

## Results

### Occurrence of *UGT33F28* mRNA variants in fall armyworm larvae

**Figure 1:**
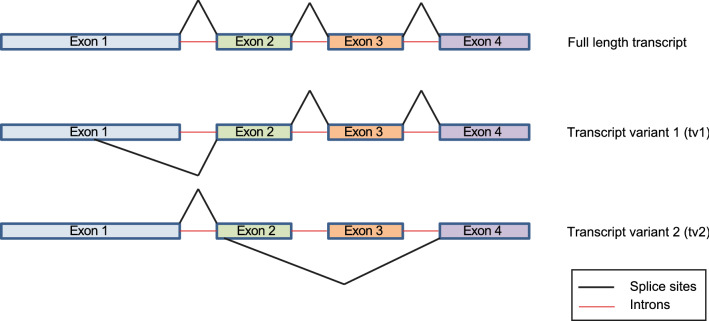
mRNA structure of full length *SfUGT33F28* transcript and variants.

Alternative forms of *UGT33F28* mRNA were identified in the midgut of fall armyworm (FAW) larvae during PCR amplification of the canonical full-length transcript. Two major variants were distinctly lower in size relative to the canonical transcript (1574 bp), and therefore harbored potential deletions resulting in truncated proteins. Upon sequencing, one transcript variant (tv1) was found to carry an internal deletion of 648 bp in exon 1, leading to an N-terminal truncation in the region responsible for substrate binding^30^ without a translational frameshift. A second variant (tv2) carried an internal deletion of 499 bp encompassing part of exon 2 and the complete exon 3, leading to a frameshift and C-terminal truncation in the sugar binding domain of the encoded protein^30^. A schematic illustration showing the splicing pattern of *SfUGT33F28* is depicted in Fig. 1.

### *SfUGT33F28* variant expression is regulated by DIMBOA

**Figure 2:**
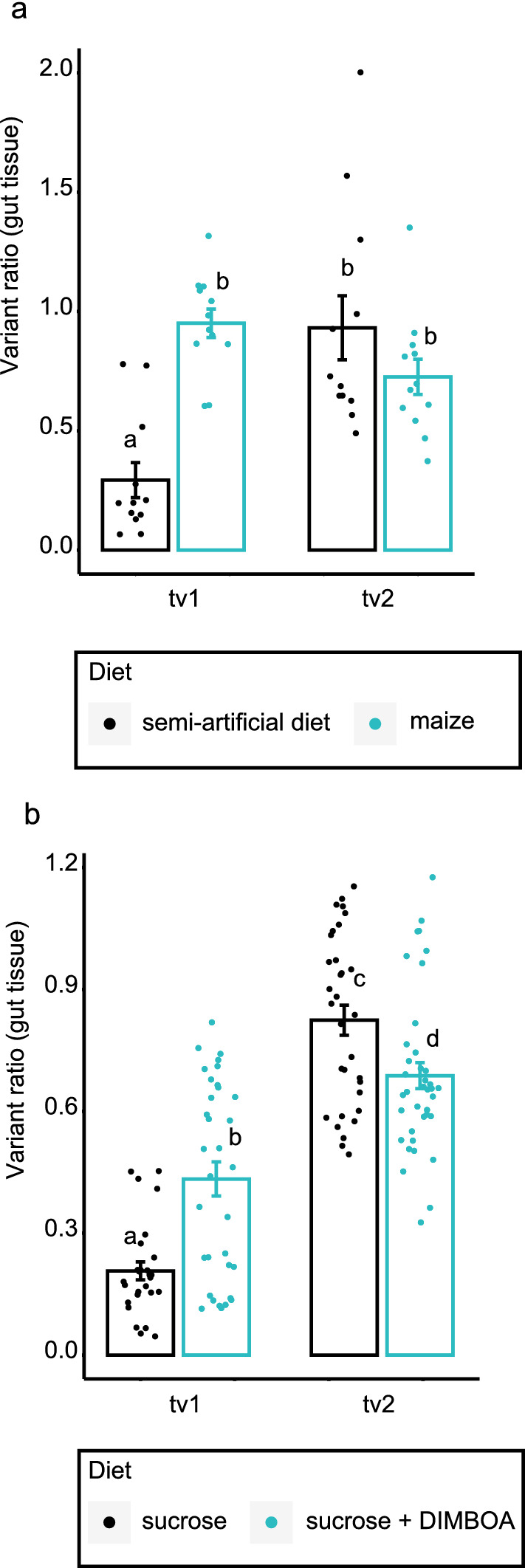
Real time PCR quantification of *SfUGT33F28* variants tv1 and tv2 in FAW larval midguts after feeding on different diets. (**a**) After feeding on semi-artificial diet compared to maize leaves, (**b**) After feeding with sucrose only or 0.625 mM DIMBOA-solution in sucrose. All expression data are represented as ratios of variant transcripts relative to the full length canonical transcript. Data are presented as mean ± SEM (n = 12–15 for semi-artificial diet versus maize feeding, n = 30–40 for feeding on sucrose solution). All values were square root transformed to meet the criteria for normality. Two-way ANOVA was performed, and the Holm–Sidak method was applied to carry out pairwise comparisons (**a**,**b**). Small letters on the bars indicate significant differences at P < 0.05.

Comparing various FAW organs (midgut, Malphigian tubules, fat bodies, cuticle) by quantitative PCR revealed that both variant mRNAs were most enriched in the larval midgut relative to the canonical *UGT33F28* transcript (Supplementary Fig. 1 and Fig. 2). When insects fed on a semi-artificial diet without DIMBOA, relative expression of tv1 (shown here as a ratio relative to full length canonical transcript) in the larval midgut was significantly lower than the relative expression of tv2 (Fig. 2a, P < 0.001). The differences between the variants were not immediately apparent in insects kept on maize only (P > 0.05). The relative expression of tv1 in the midgut, however, increased more than twofold upon switching the insects to a maize leaf diet for a day (0.293 ± 0.073 to 0.951 ± 0.06, P < 0.001). Conversely, tv2 expression was unchanged upon switching from the semi-artificial diet (0.932 ± 0.13) to maize (0.726 ± 0.073, P = 0.084) (Fig. 2a). Overall, the effects of relative transcript levels of the two variants (tv1 vs tv2, F = 6.306, P = 0.016), the diet (semi-artificial vs maize, F = 5.291, P = 0.026) and the interaction between the variants and the diet (F = 23.011, P < 0.001) were statistically significant.

In order to more clearly distinguish the effects of DIMBOA on induction of variant expression, larvae were fed with droplets of sucrose solutions with or without DIMBOA. Here again, tv1 expression increased upon exposure to DIMBOA (P < 0.001), while tv2 expression was lower in larvae ingesting DIMBOA (P = 0.005, Fig. 2b). Both the transcript levels of the two variants (tv1 vs tv2, F = 152.369, P < 0.001) and the interaction between the variants and the diet (F = 26.372, P < 0.001) were statistically significant.

### *SfUGT33F28* mRNA variants are insensitive to nonsense mediated decay

Quantitative PCR-based examination of Sf9 cultured cells (derived from *S. frugiperda* ovary cells) confirmed that insect cells also endogenously express the variant mRNA alongside the canonical transcript, albeit at different levels compared to larval guts (Supplementary Fig. 2a). Furthermore, truncated transcripts may often be recognized as “aberrant” by cells by means of mRNA surveillance and quickly degraded after synthesis. The stability of the variant *SfUGT33F28* mRNAs was thus evaluated in cultured FAW Sf9 cells using cycloheximide (CHX). In the presence of CHX, an inhibitor of nonsense mediated decay, the ratios of variant to full-length transcripts remained unaltered for both variants (P = 0.250 for tv1, P = 0.171 for tv2) over the entire duration of the experiment (Supplementary Fig. 2b,c). These results suggest that the variant mRNAs do not undergo nonsense mediated decay and could be translated alongside the full-length transcript.

### *SfUGT33F28* variable exon splicing is regulated by promoter elements within the 5′UTR

**Figure 3:**
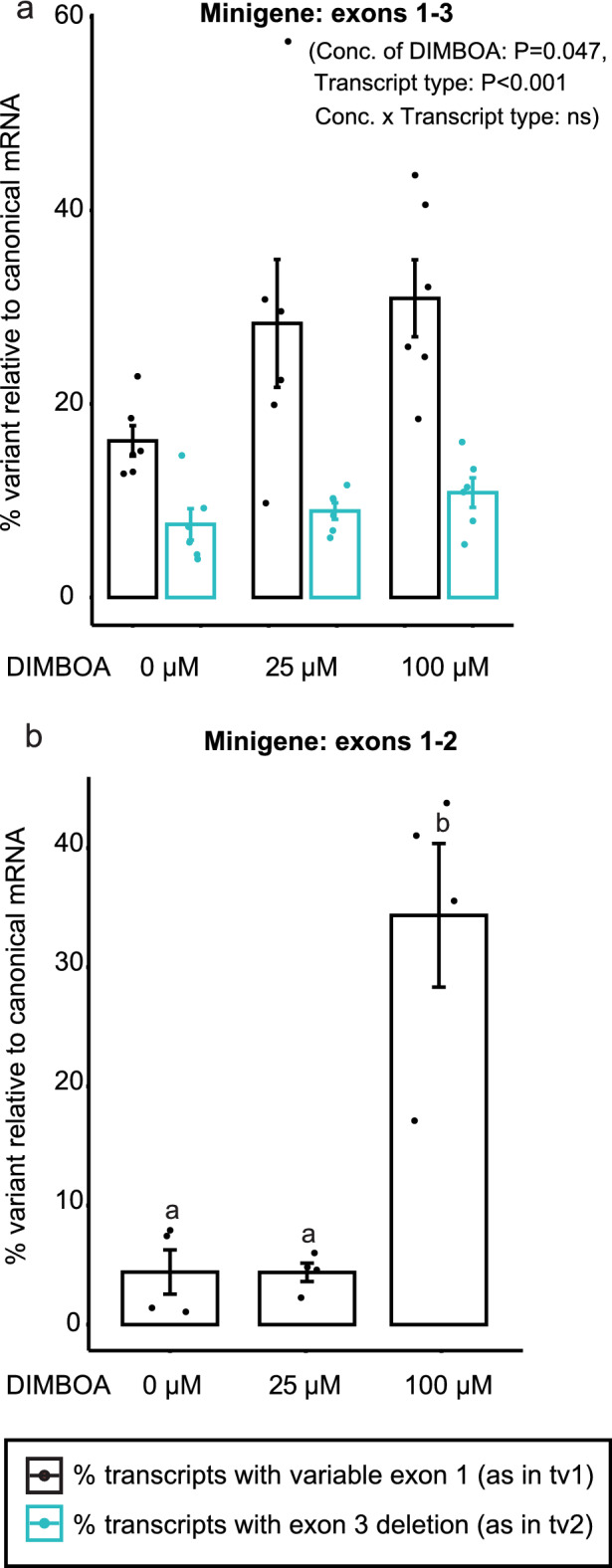
Splicing of *SfUGT33F28*-derived minigenes in *Trichoplusia ni*-derived Hi5 insect cells. (**a**) Real time PCR quantification of variable mRNA relative to the canonical *SfUGT33F28* transcripts in (**a**) the minigene containing exons 1–3 and (**b**) the minigene containing exons 1–2, with and without DIMBOA treatment. Data are represented as mean ± SEM (n = 4–6). Two-way repeated measures ANOVA was performed, and the Holm–Sidak method was applied to carry out all pairwise comparisons in (**a**), while one-way repeated measures ANOVA was performed, and the Holm–Sidak method was applied to carry out all pairwise comparisons in (**b**). Small letters on the bars indicate significant differences at P < 0.05.

To investigate the features that trigger alternative splicing, a minigene can be constructed containing a genomic DNA segment that includes the variable exon and its flanking genomic regions (encompassing at least one constitutive exon)^31,32^. The sequences regulating alternative splicing are usually located 200–300 nucleotides upstream or downstream of the variable exon^33,34^. Hence, to study the splicing pattern of *UGT33F28* we amplified a DNA fragment encompassing the promoter, 5′ untranslated (UTR) region, and the region spanning exons 1 to 3 from genomic DNA of FAW larvae, totaling 3034 bp. When introduced into *Trichoplusia ni*-derived Hi5 cultured cells, which are devoid of endogenous glycosylation activity towards DIMBOA, transcripts containing variations of both exon 1 and region spanning exon 2 and 3 could be detected after each transfection, indicating that the amplified sequence indeed carried the essential information required for alternative splicing (Fig. 3a). Both factors [concentration of DIMBOA provided in the culture medium (F = 3.783, P = 0.047) and the transcript (tv1 vs tv2; F = 35.286, P < 0.001)] were found statistically significant, while their interaction was not (F = 1.883, P = 0.186).

After addition of DIMBOA, the accumulation of transcripts with the variable exon 1 (as in tv1) increased from 12–20% (untreated) to 18–57% (P = 0.012, Fig. 3a). On the other hand, the accumulation of transcripts lacking exon 2 and 3 (as in tv2) remained in the range of 4–16%, regardless of DIMBOA treatment (P > 0.05, Fig. 3a).

In order to remove the influence of exon 3, we designed another minigene construct to study the variable splicing of exon 1 in isolation, which encompassed the *SfUGT33F28* promoter, 5′ UTR, and the region spanning exons 1 and 2, totaling 2348 bp. After introduction into Hi5 cells, the cells were subjected to treatment with DIMBOA in the same way as described above and a significant induction of transcript expression was observed (F = 26.340, P = 0.001 between groups). The percentage of mRNA containing the exon 1 variant (as in tv1) increased nearly eightfold upon treatment of the cells with 100 µM DIMBOA, an average induction of 20–40% as a proportion of all *SfUGT33F28* transcripts (P = 0.002, Fig. 3b). Thus, analysis of these minigene constructs in insect cells established that the variable splicing of exon 1, which is regulated by DIMBOA, is due to genomic sequence elements flanking the *SfUGT33F28* coding region.

### An insect aryl hydrocarbon receptor motif mediates variable splicing of *UGT33F28*

**Figure 4:**
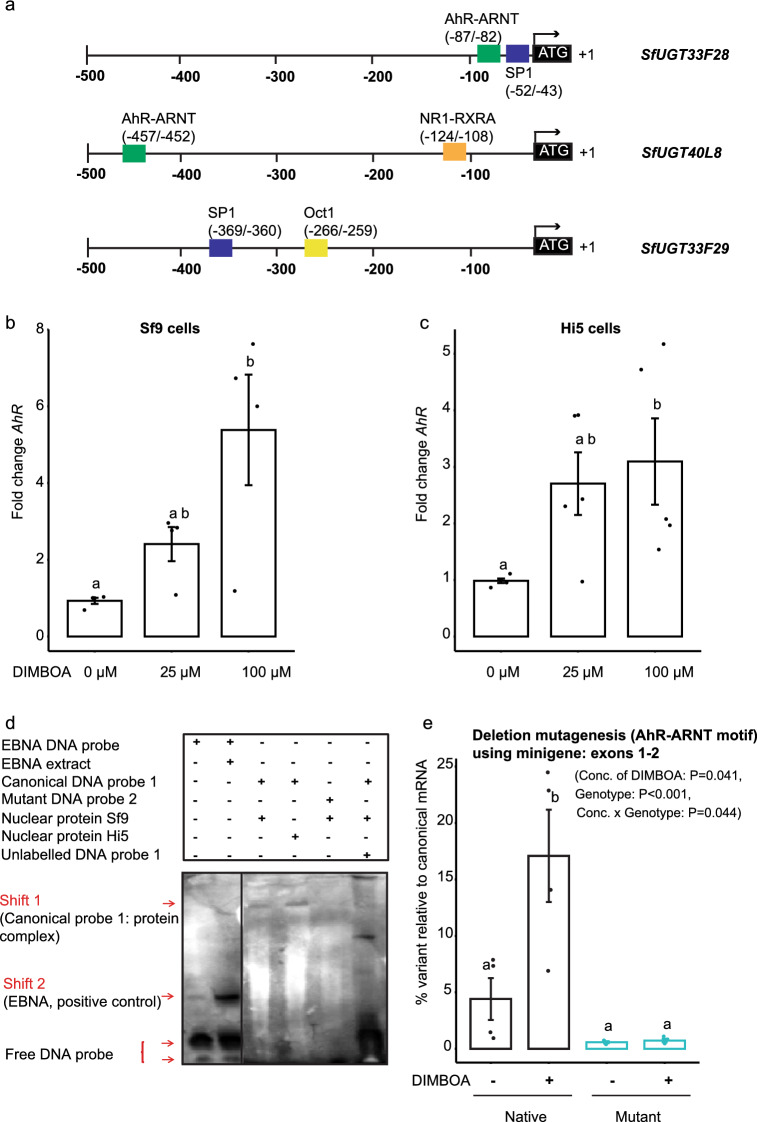
Aryl hydrocarbon receptor (AhR) regulates formation of *SfUGT33F28* transcript variants. (**a**) Schematic depiction of 5′ promoter elements in SfUGTs (*AhR* aryl hydrocarbon receptor, *ARNT* aryl hydrocarbon receptor nuclear translocator, *SP1* specificity protein 1, *NR1/RXRA* nuclear receptor 1/retinoid X receptor A, *Oct1* Octamer 1). (**b**,**c**) Quantitation of *AhR* transcript levels in DIMBOA-treated Sf9 and Hi5 insect cells by real time PCR (n = 4–5). (**d**) Electrophoretic mobility shift assay (EMSA) to demonstrate the binding of the AhR-ARNT protein complex from insect cells to the nucleotide sequence derived from *SfUGT33F28* 5′ UTR (canonical DNA probe). The mutant DNA probe lacked the same nucleotide sequence. As a positive control, the Epstein-Barr Nuclear Antigen (EBNA) was demonstrated to bind to a biotin-EBNA probe. (**e**) Deletion mutagenesis of the AhR-ARNT DNA binding motif in the *SfUGT33F28* exon 1–2 minigene. The graph depicts the RT-PCR quantification of variant mRNA relative to the canonical *SfUGT33F28* mRNA after transfection of Hi5 cells with the native minigene and compared to the deletion mutant with or without DIMBOA treatment (n = 4). All data are represented as mean ± SEM. One-way ANOVA on ranks was performed (**a**) and Tukey’s test was applied to carry out all pairwise comparisons in (**a**) and (**b**). Two-way repeated measures ANOVA was performed, and Holm–Sidak method was applied to carry out all pairwise comparisons in (**e**). Small letters on the bars indicate statistically significant differences at P < 0.05.

We next analyzed the sequences flanking exon 1 in more detail, searching for potential transcription factor binding motifs that might regulate *SfUGT33F28* splicing. The sequence upstream of exon 1 (utilized in the preparation of *UGT33F28* minigene) showed identity to a few well-defined transcription factor binding motifs in the JASPAR database^35^, including 100% conservation with a response element of the human UGT2B10 gene for a protein complex consisting of the aryl hydrocarbon receptor (AhR) and the aryl hydrocarbon receptor nuclear translocator (ARNT). Promoter analyses using *SfUGT40L8*, another UGT capable of glucosylating DIMBOA but expressed at low levels in the FAW^15^, also indicated the presence of a distal AhR-ARNT binding motif. On the other hand, the closest relative of *SfUGT33F28*, *SfUGT33f29*, which does not show any activity towards DIMBOA or other benzoxazinoids did not harbor the motif (Fig. 4a). In addition to the AhR-ARNT binding motif, binding sites were found in these SfUGTs for other transcription factors previously found to participate in xenobiotic detoxification in insects (Fig. 4a).

As benzoxazinoids, such as DIMBOA, are indole-derived aromatic compounds, we hypothesized that DIMBOA could serve as a potential ligand for this receptor. The putative AhR-encoding gene in *S. frugiperda* was identified from the published *S. frugiperda* transcriptome database^36,37^ based on protein sequence similarity to known AhRs using *D. melanogaster* and *S. litura* sequences as queries. SfAhR was found to show a strong conservation with known and predicted AhR protein sequences across the class Insecta (90–100% similarity, Supplementary Table 3). Similar searches performed using the published *S. frugiperda* transcriptome yielded a putative *ARNT* gene in *S. frugiperda* (GSSPFG00020774001). The *S. frugiperda* ARNT protein also showed a strong similarity across insects (in the range of 75–100%, Supplementary Table 4). Much like its characterized counterparts, the lepidopteran ARNT protein was highly conserved in the N-terminus region responsible for protein dimerization with AhR and carried monopartite nuclear localization signals (NLS), suggestive of potential translocation to the nucleus (Supplementary Table 5).

The expression of *AhR* was induced ~ 6-fold in Sf9 cells (H = 8.465, P = 0.002 between groups) and ~ 3-fold in Hi5 cells (F = 4.241, P = 0.04 between groups) by exposure to 100 µM DIMBOA when compared to untreated cells, providing support for the involvement of the AhR receptor in DIMBOA-induced variable splicing (Fig. 4b,c). However, expression of *ARNT* did not respond to treatment with DIMBOA (Supplementary Fig. 3a,b). We tested the specificity of the *AhR* response using other polyaromatic plant defense compounds including indole (a benzoxazinoid precursor), quercetin (a flavonoid) and esculetin (a coumarin), which had been previously shown to elicit AhR-mediated responses^38^. *AhR* expression was significantly up-regulated up to fivefold in response to treatment with indole (P = 0.004), while quercetin led only to a weak (twofold) up-regulation (P = 0.165) (Supplementary Fig. 3c). Esculetin, on the other hand, led to a mean 3–4 fold up-regulation of *AhR* expression, albeit with a high variability (P = 0.319).

To investigate the binding of the AhR:ARNT protein complex with the putative DNA binding motif in the 5′ UTR of the *SfUGT33F28* gene, we performed an electrophoretic mobility gel shift assay (EMSA). A short biotinylated DNA probe encompassing the 6 nt candidate motif was found to bind to the nuclear protein fraction extracted after treatment of insect cells with DIMBOA, as evidenced by a shift in the migration of the probe, when compared to the lane containing the probe without the nuclear protein fraction (Fig. 4d, Supplementary Fig. 4). A mutated DNA probe lacking the motif, however, was not bound by the nuclear protein fraction and did not undergo an electrophoretic shift. Streptavidin agarose purification of the proteins bound to the biotinylated DNA probe resulted in the detection of AhR by proteomic analyses (Supplementary Table 8), supporting the participation of this aryl hydrocarbon receptor in DIMBOA-induced variable splicing in the FAW.

To confirm the role of the conserved AhR-ARNT motif in alternative splicing of the *SfUGT33F28* gene, we mutagenized the corresponding region of the originally made *SfUGT33F28* minigene spanning exons 1 and 2. Both factors (genotype, F = 25.356, P = 0.002; and concentration of DIMBOA, F = 6.738, P = 0.041) as well as their interaction (F = 6.444, P = 0.044) played a significant role. When the 6 nt putative AhR-ARNT motif (nucleotides − 82 to − 87 relative to the start site) was deleted, the frequency of the mRNA variant containing the exon 1 truncation declined from 4.4% for cells transfected with the original exon 1–2 minigene construct to 0.58% for cells transfected with the mutant minigene carrying the motif deletion (Fig. 4e). Upon treatment with DIMBOA, the frequency of the variant increased in cells transfected with the native minigene increased to 17.1% (P = 0.011) but cells transfected with the mutant minigene showed a reduction for variant expression to 0.72% (P < 0.001). Thus, deletion of the AhR-ARNT motif drastically reduced the DIMBOA-inducible expression of *UGT33F28*, indicating that this response element is critical for the activation of *UGT33F28*.

### The proteins produced from *SfUGT33F28* transcript variants are catalytically inactive, but alter the rate of DIMBOA glucosylation

**Figure 5:**
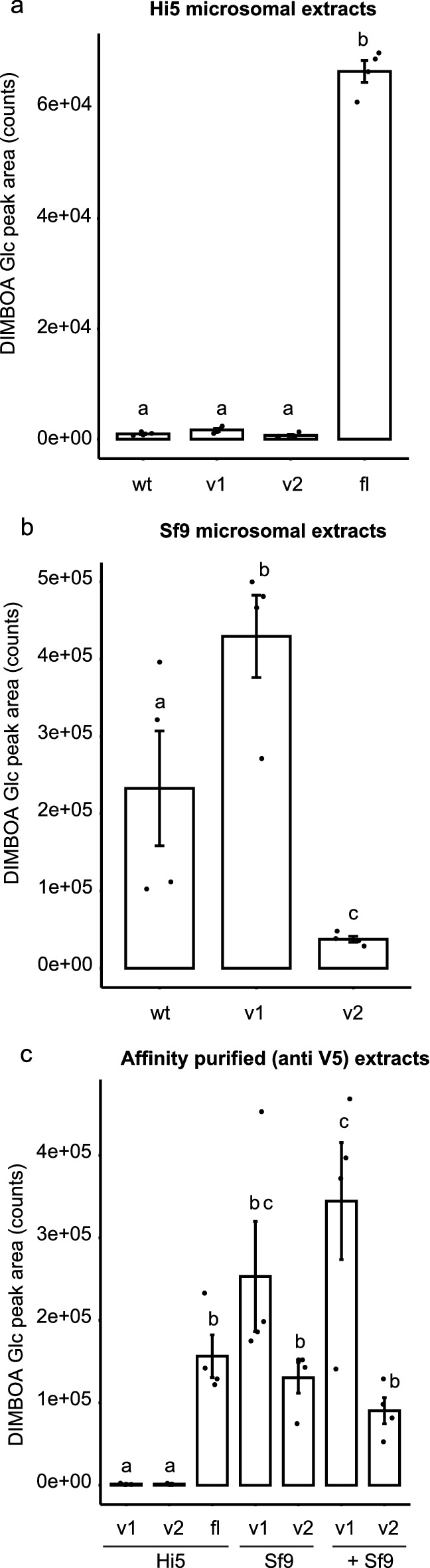
Truncated SfUGT33F28 variant proteins are catalytically inactive but may interact with the full-length UGT33F28 protein. (**a**,**b**) In vitro enzymatic assays with microsomes derived from Hi5 cells and Sf9 cells over-expressing SfUGT33F28 variants towards DIMBOA as substrate, (**c**) in vitro enzymatic assays with affinity-purified microsomes derived from Hi5 cells and Sf9 cells over-expressing SfUGT33F28 variants using DIMBOA as substrate. Data are represented as mean ± SEM (n = 4). One-way repeated measures ANOVA was performed and the Holm–Sidak method was applied to carry out all pairwise comparisons. Small letters on the bars indicate significant differences at P < 0.05. *wt* wildtype, *v1* variant 1, *v2* variant 2, *fl* full-length.

To characterize the proteins encoded by the variant *SfUGT33F28* transcripts, we heterologously expressed them in two lepidopteran insect cell lines, Hi5 and Sf9. The Sf9 line was included since it possesses a native UGT activity for DIMBOA^15^, and thus would help evaluate whether the truncated proteins correctly underwent any post-translational modifications necessary for catalysis. Western blot analysis established the presence of a ~ 15 kDa protein resulting from the expression of variant 1 (from tv1) and a ~ 40 kDa protein resulting from the expression of variant 2 (from tv2) in both cell lines, as would be predicted from their mRNA sequences. These bands were found in microsomal extracts of the cells, but not in the media and the cytoplasmic fractions obtained during microsome preparation. Hi5 cells expressing the variant proteins from tv1 (UGT33F28_v1) and tv2 (UGT33F28_v2) showed no detectable UGT activity towards DIMBOA, but this activity was observed in Hi5 cells producing full length UGT33F28 protein (F = 1105.209, P < 0.001 between groups; Fig. 5a). Surprisingly, Sf9 cells producing the UGT33F28_v1 displayed an overall increase in their DIMBOA glucosylation specific activity compared to untransfected Sf9 cells, while Sf9 cells producing the UGT33F28_v2 variant showed a decreased DIMBOA glucosylation activity (F = 18.275, P = 0.003 between groups; Fig. 5b).

To better understand the changes in the in vitro UGT activities of Sf9 cells producing the truncated variants, affinity purification was carried out taking advantage of the V5 tags present in the heterologously produced proteins. Affinity-purified protein fractions from Sf9 cells producing the variant proteins retained their activity towards DIMBOA despite the lack of intrinsic UGT activity displayed by these truncated variants when produced in Hi5 cells (F = 11.825, P < 0.001 between groups; Fig. 5c). Furthermore, we observed that microsomes derived from Hi5 cells expressing the variant protein gained DIMBOA-UGT activity after being trapped on anti-V5 resin and co-incubated with microsomes from untransfected Sf9 cells. This strongly suggested that the truncated isoforms containing V5 tags had become bound to the DIMBOA-UGT enzymes already present in Sf9 cells, which do not contain a V5 epitope and thus do not bind to the resin by themselves.

### SfUGT33F28 variant proteins may form oligomers with the full-length SfUGT33F28 protein

The unexpected results of our assays of heterologously-expressed variant proteins motivated us to determine if FAW UGTs in insect cells exist in an oligomerized state and whether variant and full-length proteins could be bound as hetero-oligomers. Dimethylsuberimidate (DMS) was employed as the cross-linking agent because it can react with primary amines over a wide pH range^39^. Cross-linking assays with the full-length UGT33F28 protein, followed by western blot analyses resulted in bands corresponding to 55 kDa (monomer), 110 kDa (likely a homodimer) and ~ 250 kDa (Supplementary Fig. 5). Cross-linking assays with Sf9 cells expressing UGT33F28_v1 against a background of endogenous UGT activity on DIMBOA yielded bands of ~ 15–20 kDa (variant 1 monomer) and a higher oligomer at ~ 70 kDa (Supplementary Fig. 5) suggesting the existence of a heterodimer formed between the variant and the full-length UGT protein.

Similarly, cross-linking of Sf9 cells expressing UGT33F28_v2 followed by SDS-PAGE and western blot analyses resulted in bands corresponding to the expected variant 2 monomer at ~ 35–40 kDa, as well as a ~ 100 kDa band matching the size of a heterodimer between the variant and the full-length UGT protein (Supplementary Fig. 5). These results thus support the possibility that the truncated UGT33F28 proteins derived from the transcript variants exist in oligomeric forms with full-length UGT33F28.

### UGT33F28 variants co-purify with the full-length protein and modulate its DIMBOA glucosylation activity

To further investigate the interactions of SfUGT33F28 variant proteins with the full-length UGT33F28 suggested by the cross-linking experiments, we affinity-purified V5-tagged SfUGT33F28 variants and analyzed their binding partners by proteomics (Supplementary Fig. 6a). These analyses identified a range of likely candidates binding with the variant proteins. The variant protein UGT33F28_v1 was found to co-purify with full-length SfUGT33F28, as well as SfUGT40R12, SfUGT40R13 and SfUGT40F19 (Supplementary Table 9), while none of these proteins were be detected in the resin flow-through. A number of other microsomal proteins also co-purified with UGT33F28_v1, most noticeably CYP9A30, which belongs to a CYP family highly induced in response to plant allelochemicals^38^. Similar analyses performed with UGT33F28_v2 revealed that SfUGT33F28, SfUGT33T9, SfUGT33S2, and several microsomal proteins (Supplementary Table 10) including CYP314A1 and carboxylesterase 021c co-purified with the variant protein.

**Figure 6:**
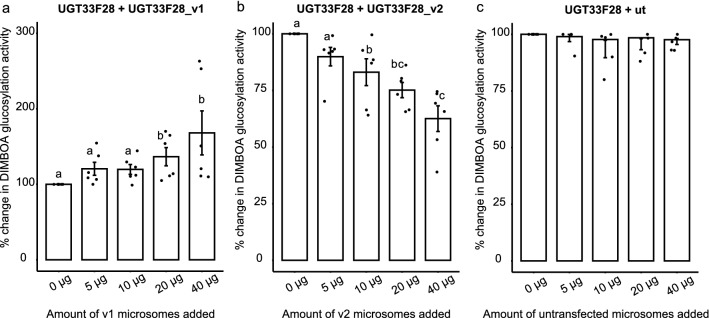
UGT33F28 variants modulate the DIMBOA glucosylation activity of the full-length protein. (**a**–**c**) In vitro enzymatic assays with microsomes from Hi5 cells producing SfUGT33F28 incubated with increasing amounts of microsomes from Hi5 cells producing SfUGT33F28 variant proteins towards DIMBOA as substrate. Microsomes from untransfected Hi5 cells (ut), which showed no activity towards DIMBOA, were used as a control and added in the same amount as the variant microsomes. DIMBOA-Glc peak areas were converted to relative activities with activity of the full-length protein set at 100%. Data are represented as mean ± SEM (n = 6). One-way repeated measures ANOVA was performed and the Holm–Sidak method was applied to carry out all pairwise comparisons. Small letters on the bars indicate a significant difference at P < 0.05. *v1* variant 1, *v2* variant 2.

The UGTs found to bind to the variant proteins were then heterologously produced in *T.ni* cells (Supplementary Table 11). Among the UGTs detected via proteomic analyses, only SfUGT33F28 had been previously shown to glycosylate DIMBOA efficiently. However, SfUGT40R13 and SfUGT40F19 showed low activities towards MBOA and DIMBOA respectively^15^, while SfUGT40R12 had no detectable activity towards benzoxazinoids. Since protein–protein interactions within the UGT family can modify the activities of these proteins^23,27^, combinatorial assays were performed to test how each of the variants affected the catalytic activities of these UGTs towards DIMBOA. These assays used extracts obtained from Hi5 cells producing UGT33F28_v1 together with extracts from Hi5 cells producing either SfUGT33F28, SfUGT40R12, SfUGT40R13 or SfUGT40F19, respectively (Supplementary Fig. 6b). Incubation of variant 1 with the full length UGT33F28 protein led to a marked increase in DIMBOA glucosylation activity, while all other combinations gave no observable change in activity. Subsequent incubations of the full-length UGT33F28 protein with DIMBOA in the presence of increasing amounts of variant 1 gave an increase in DIMBOA glucosylation activity up to 50% more than that shown by the full-length protein alone (Fig. 6a, P = 0.006 between groups).

Combinatorial assays of DIMBOA glucosylation activity were also performed with UGT33F28_v2 and the corresponding UGTs that co-purified with it based on proteomic analyses. Extracts from Hi5 cells producing UGT33F28_v2 were incubated in separate reactions with Hi5 cells producing either SfUGT33T9, SfUGT33S2 or SfUGT33F28, respectively. SfUGT33T9 and SfUGT33S2 were found to have no DIMBOA-UGT activity on their own, or in combination with the variant 2 protein. SfUGT33F28, on the other hand, was only slightly influenced by the presence of UGT33F28_v2 (Supplementary Fig. 6c). Following up on these preliminary results, full-length UGT33F28 protein was incubated with increasing amounts of the variant 2 protein, resulting in a decrease in DIMBOA glucosylation activity up to 30% (Fig. 6b, P < 0.001 between groups). No changes in activity were noted when equivalent amounts of untransfected Hi5 microsomes were added to the full-length UGT33F28 protein as a control (Fig. 6c, P > 0.05 between groups).

## Discussion

UDP glycosyltransferases (UGTs) are a superfamily of enzymes present in all kingdoms of life that transfer sugar residues to a wide range of small, often lipophilic acceptors. In insects, most UGTs use UDP-glucose as a donor and play roles in diverse processes such as detoxification, olfaction, pigmentation and cuticle formation. However, how UGT activity is regulated is poorly known. We sought to learn more about the molecular players underlying the regulation of detoxification of DIMBOA, a major anti-herbivore maize defense compound which is detoxified via UGT33F28-mediated glucosylation in the fall armyworm (FAW, *Spodoptera frugiperda*)^15,16^.

In this work, we discovered a series of transcript variants of the corresponding gene *SfUGT33F28* that display large deletions in their coding regions, leading to the formation of truncated proteins. Transcript variations in human UGTs have been previously reported that cause complete skipping of exon 1, the N-terminal aglycone binding domain, or deletions in the exons that encode the C-terminal co-substrate domain^22^. The present work showed that such alternative forms of *UGT33F28* mRNA are expressed in the FAW as well. Interestingly, the ratio of the expression of these UGT variants relative to the canonical transcript reflected shifts in larval diet. For example, *SfUGT33F28* transcript variant 1, with a deletion of exon 1, was expressed in the larval midgut at higher levels relative to the canonical transcript when FAW fed on maize with its defense compound DIMBOA. This observation was corroborated when larvae were orally administered DIMBOA. Accordingly, we investigated the mechanisms underlying the expression of these variants and their consequences for UGT activity against DIMBOA.

In higher eukaryotes, alternative splicing plays a major role in generating transcript diversity, which can be constitutive or induced in response to abiotic and biotic stimuli^40–42^. In order to assess if alternative splicing events were responsible for the generation of the *UGT33F28* variants, we employed Sf9 cells, originally derived from FAW, which have an endogenous ability to glycosylate DIMBOA. Furthermore, cultured insect cells provided a good tool in vitro to assess whether such truncated transcripts could undergo translation. Sf9 cell treatment with cycloheximide did not lead to super-induction of *UGT33F28* variants, implying that the variant transcripts are stable and could undergo translation. Furthermore, not only were the variant transcripts of *UGT33F28* stable, but the alternative splicing events creating them were shown to arise directly from sequence elements of the *UGT33F28* gene. The *T. ni* derived Hi5 cell line, which is devoid of DIMBOA glucosylation activity was transfected with a *UGT33F28* minigene containing only the promoter, 5′ UTR and exons 1 and 2. When these cells were exposed to DIMBOA, we observed an increase in transcripts with the variable exon 1 relative to the canonical transcript.

In the search for what sequences in the 5′ UTR region mighty trigger this transcript diversity, a classic xenobiotic response element was found, the AhR-ARNT motif. AhR belongs to the basic helix–loop–helix/Per-Arnt-Sim (bHLH/PAS) family of receptors and has been previously characterized in response to an array of plant toxins and other xenobiotics including polycyclic aromatic hydrocarbons such as benzopyrene^43^, the furanocoumarin xanthotoxin^44^, gossypol^45^, and tryptophan derivatives such as indole [3, 2-*b*] carbazole (ICZ)^46^ and serotonin^47^. Upon ligand binding, the AhR complex can direct the transcription of several detoxification genes—most notably phase I detoxification enzymes, such as the cytochrome P450s, and phase II detoxification enzymes, such as glutathione-*S*-transferases, UGTs and NADPH oxidoreductases^48,49^. Furthermore, the AhR-ARNT motif of *SfUGT33F28* was very similar to those in mammalian genes that are known to be targets of aryl hydrocarbon receptor regulatory cascade(s), suggesting that a regulatory process inducing the expression of insect and vertebrate phase II detoxification genes might be evolutionarily conserved. Accordingly, we observed that the *AhR* transcripts were highly induced in the very first hour of DIMBOA treatment. Previous studies have reported that the AhR-ARNT receptor has a very short half-life, leading to loss of active AhR-ARNT complexes and the quick attenuation of the response towards the inducing xenobiotic^50^. We confirmed the role of this receptor in *UGT33F28* activation by deleting the AhR-ARNT response element in the 5′ UTR of *SfUGT33F28*, which led to the loss of DIMBOA-mediated regulation of the gene and a lowered incidence of transcripts with a variable exon 1. These results were also in agreement with those from the cycloheximide assay, allowing the inference that treatment with cycloheximide led to loss of a de novo protein synthesis event involving early acting AhR-ARNT transcription factors, ultimately leading to impaired *SfUGT33F28* activation. It is further plausible that AhR could interact with constitutive cell specific transcription factors, and this might account for some of the differences in expression of *UGT33F28* variants observed across larval tissues tested in this study.

Heterologous expression and functional characterization of the variant *SfUGT33F28* UGTs in *T. ni* derived Hi5 cells showed that the variants were themselves inactive towards DIMBOA. However, the expression of the variant proteins in *S. frugiperda*-derived Sf9 cells, showed unexpectedly that *UGT33F28_v1* expression elevated the rate of glucoside formation compared to wild type Sf9 cells, but that *UGT33F28_v2* expression decreased glucoside formation. These results hinted at oligomerization between the variants and the endogenous full-length UGT enzymes, and indeed the literature contains several reports of oligomerization among UGTs that result in modulation of activity or even gain of novel enzymatic activities^26,27,51,52^. Cross-linking experiments with cells expressing the variants suggested higher order complex formation, including potential heterodimer formation between the variants and other UGTs. Homodimers of the variants, however, were not observed. Previous reports indicate that UGTs with N-terminal mutations or partial deletions (such as UGT33F28_v1) are unable to engage in homodimer formation, although UGTs harboring partial C-terminal truncations (such as UGT33F28_v2) could still form homodimers^39^, partly explaining the results of our cross-linking experiments. While the exact physical nature of the protein interactions in the present study is unclear, UGTs can form covalently cross-linked complexes via intermolecular disulfide linkages^53,54^. Other protein features such as the asparagine residues that serve as potential N-glycosylation sites could also lead to structural differences^55^ that could affect protein–protein interaction and the degree of oligomerization, a topic worthy of future investigation.

Protein purification and subsequent proteomic analyses confirmed that the full length SfUGT33F28 protein indeed interacted with the UGT33F28 protein variants. Even more interestingly, when variant proteins were supplied in different amounts to the full-length UGT33F28, it became clear that variant 1 enhanced DIMBOA glucosylation activity while the variant 2 repressed it. Previous work on mammalian UGTs has demonstrated how UGT isoforms, inactive alone, can bind to full-length active isoforms and regulate their activity. However, full-length protein activities are usually repressed^56^, rather than enhanced^22^, although some heterodimers become active towards compounds that are not substrates for either of the corresponding monomers^57^.

In the present case, the expression of the *SfUGT33F28* variants could help regulate DIMBOA glucosylation activity in FAW cells in the presence and absence of DIMBOA. Without DIMBOA, the ratio of transcript variant 2 to the canonical transcript was always found to be higher than the ratio of variant 1 to the canonical transcript. Upon DIMBOA exposure, variant 1 accumulates in the cells promoting DIMBOA detoxification. As this mechanism appears to involve both newly translated and existing UGT33F28 proteins, it may be faster and more reversible than simple control of a single catalytic protein at the transcriptional level. However, further research is needed to substantiate this hypothesis. UGT activity has previously been shown to be regulated at the transcription level or via post-transcriptional regulation mediated by miRNAs^56^. The UGT33F28 variants could also interact with other UGT proteins^53^ or even other microsomal proteins that co-localize with these UGTs leading to formation of metabolons, and so to an overall increase in detoxification efficiency by coordination between phase I and phase II enzymes.

In summary, splicing variation in the detoxification gene transcripts of insect herbivores that are induced by plant defense compounds may provide an important mechanism for regulation of detoxification activity in these organisms. Given the metabolic costs of detoxification^58^, these processes may have to be closely regulated to maximize benefits to the organism. Additional investigations on detoxification enzymes should further our understanding of how these catalysts dictate host preferences and account for the ability of some insects to become serious agricultural pests.

## Materials and methods

### Insects and plants

Larvae of fall armyworm (FAW, *Spodoptera frugiperda*) were cultured at the Department of Entomology at the Max Planck Institute for Chemical Ecology, and reared on a semi-artificial diet based on pinto bean^59^, and maintained under controlled light and temperature conditions (12:12 h light/dark, 21 °C).

### Feeding experiments

3rd–4th instar FAW larvae were utilized for all experiments. Insects were starved overnight prior to feeding experiments. The following day insects were fed with a semi-artificial, pinto bean-based diet or put on maize leaves in small plastic cups and allowed to feed on the respective diets for a day. Insects were dissected in cold phosphate buffered saline (PBS, pH = 7.4) to harvest larval tissues (guts, Malphigian tubules, fat bodies, cuticle), which were stored at − 80 °C until further use. For droplet feeding, 12.5 mM DIMBOA was prepared by dissolving the compound in DMSO. This DIMBOA solution was further diluted in 10% aqueous sucrose solution. The larvae were stimulated with forceps to encourage regurgitation, and 2 μL DIMBOA-sucrose solution was administered directly to the larval mouthparts. Insects were then fed on semi-artificial diet for up to 6 h; following which gut tissue was dissected using cold phosphate buffer and the tissue samples were stored at − 80 °C until further use.

### Insect cell cultures

*Spodoptera frugiperda* Sf9 cells and *Trichoplusia ni* Hi5 cells were cultured in Sf-900 II serum-free medium (Gibco) and ExpressFive serum-free medium (Gibco), respectively. Adherent cultures were maintained at 27 °C, and sub-cultured every 3–4 days.

### Cell treatments

Insect cells were seeded in 6 well culture plates (Corning) and left at 27 °C overnight. For transcript stability tests, a fresh cycloheximide (CHX) stock (50 mg/mL) was prepared in ethanol and added to the cultured cells at a concentration of 50 µg/mL. Incubations with CHX were performed up to 6 h. For testing substrate specificity, cells were then treated with the following compounds for 1 h—DIMBOA (25–100 μM), indole (50–100 μM), quercetin (50–100 μM), and esculetin (50–100 μM). All the stocks were prepared in DMSO and cells treated with the corresponding volume of pure DMSO served as a control. The range of concentrations used for the substrates was based on previous work^38^.

### RNA extraction, reverse transcription and real time-PCR analysis

Tissue samples from the larvae were homogenized and total RNA extracted using the innuPREP RNA Mini Kit (Analytik Jena). Cell cultures used for RNA extraction were obtained during sub-culturing at full confluency, and centrifuged at 500×*g* for 5 min. The culture medium was discarded, and the fresh pellets were directly used for RNA extraction. RNA concentrations were measured with the NanoDrop 2000 UV–Vis Spectrophotometer (Thermo Scientific). First strand cDNA was synthesized from 1 μg total RNA using SuperScript III Reverse Transcriptase and OligodT primers from Invitrogen. Sequences were successfully amplified using Phusion High Fidelity DNA Polymerase (New England Biolabs) (PCR protocol: 30 s at 98 °C; 35 cycles of 10 s at 98 °C, 20 s at 55 °C, 45 s at 72 °C; and 5 min at 72 °C). The PCR products were purified with a PCR cleanup kit (Qiagen) and cloned into pCR-Blunt II-TOPO vector (Life Technologies) and transformed into NEB cells (Life Technologies), which were plated on selective LB agar medium containing 100 μg/mL ampicillin and incubated overnight at 37 °C. Positive colonies were identified by PCR using vector-specific M13 primers. Positive clones were confirmed by sequencing. Real time PCR analyses were carried out using Brilliant III SYBR Master Mix, employing SYBR Green chemistry. Relative quantification of the transcript levels was done using the 2^−∆∆Ct^ method^60^. *SfRPL10* was used as reference gene for all analyses. The primer pairs used for distinguishing between the variants are listed in Supplementary Table 1. As the expression of full-length and variants of *SfUGT33F28* differed according to the strains, tissues, and treatments being analyzed, variant expression is reported as ratios relative to the canonical transcript to facilitate comparisons.

### Preparation of minigenes for alternative splicing studies

Genomic DNA was isolated from *S. frugiperda* larvae using the cetyl trimethyl ammonium bromide (CTAB) protocol^61^. DNA concentration was measured with the NanoDrop 2000 UV–Vis Spectrophotometer (Thermo Scientific). The minigene was amplified using Phusion High Fidelity DNA Polymerase (New England Biolabs) (PCR protocol: 30 s at 98 °C; 35 cycles of 10 s at 98 °C, 30 s at 55–60 °C, 1 min 30 s at 72 °C; and 10 min at 72 °C), cloned into a pCR-Blunt II-TOPO vector (Life Technologies) and sequenced using M13 primers. The confirmed sequence was eventually cloned into a pIB/V5-His-TOPOvector (Life Technologies) and transformed into NEB cells (Life Technologies). Positive colonies were identified by colony PCR using vector-specific *OpIE2* primers, sub-cultured overnight at 37 °C in liquid LB medium containing 100 μg/mL ampicillin and used for plasmid DNA purification with the NucleoSpin Plasmid kit (Macherey-Nagel). Concentration and purity of the obtained construct was assessed by the NanoDrop 2000 UV–Vis Spectrophotometer (Thermo Scientific) and the correct orientation of the PCR products was confirmed by DNA sequencing.

### Nuclear protein isolation

Nuclear proteins were isolated from insect cells^62^ using the protocol originally described with few modifications. Cells grown to concentrations of up to 1 × 10^6^ cells/well were harvested and washed with PBS (pH 7.4). The extracts were centrifuged at 12,000×*g* for 10 min and pellets were re-suspended in 400 μL cell lysis buffer (10 mM HEPES, pH 7.5, 10 mM KCl, 0.1 mM EDTA pH 8.0, 1 mM DTT, 0.5% Nonidet-40 and 10 μL protease inhibitor cocktail). Cells were allowed to swell on ice for 20 min with intermittent mixing. Suspensions were vortexed to disrupt the cell membranes and then centrifuged at 12,000×*g* for 10 min at 4 °C. Pelleted nuclei were washed thrice with cell lysis buffer, re-suspended in 50 μL nuclear extraction buffer (20 mM HEPES pH 7.5, 400 mM KCl, 1 mM EDTA pH 8.0, 1 mM DTT, 10% glycerol and protease inhibitor) and incubated on ice for 30 min. Nuclear fractions were collected by centrifugation at 12,000*g* for 15 min at 4 °C. Protein concentrations were measured by Bradford and extracts were stored at − 80 °C until further use.

### Electrophoretic mobility shift assay (EMSA)

EMSA was performed using the LightShift Chemiluminescent EMSA kit (Thermo Scientific) following the manufacturer’s instructions. Genomic DNA fragments of 20–25 bp corresponding to the 5′ flanking region of UGT33F28 exon 1 (with and without AhR-ARNT motif deletion) were synthesized with covalently linked biotin (Sigma). The DNA probes used in the experiment are listed in Supplementary Table 6. EMSA was performed in 20 µL reactions containing 20 fmol biotinylated DNA probe with 3.5–4 µg nuclear protein extracted from insect cells, according to manufacturer’s instructions. A reaction comprising the above along with the excess of unlabeled canonical DNA probe (200 molar excess) was further employed as a control. The reaction was assembled at room temperature and incubated for 30 min. The reactions were separated on a 5% TBE gel in 0.5X TBE at 100 V for 60 min. The samples were then transferred to a positively charged nylon membrane (Hybond N^+^, Amersham Bioscience) using semi-dry transfer at 15 V for 30 min. The membrane was cross-linked for 1 min using the auto cross-link function on the UV cross-linker (Stratagene). The biotinylated DNA–protein complex was detected by the streptavidin–horseradish peroxidase conjugated antibody provided in the kit. The membrane was washed and incubated with the chemiluminescence substrate for 5 min and the signals were developed by exposing the membrane to an X-ray film for 1 min.

### Streptavidin affinity purification

Streptavidin agarose (Sigma-Aldrich) was employed for protein purification. Briefly, 50–100 μL of agarose was packed into a 1.5 mL Eppendorf tube for each sample. The agarose was allowed to settle with a short centrifugation (500×*g*, 5 min) and the supernatant was discarded. The agarose was washed 4–5 times with binding buffer (PBS containing 1 mM EDTA, 1 mM DTT, 4 µg poly dI. dC as non-specific competitor DNA and protease inhibitor). Simultaneously, the binding reaction with the nuclear protein fraction and the DNA probe was assembled as described above. A 100 μg amount of total nuclear protein was incubated with 4 μg of biotinylated DNA probe at room temperature for 20 min. The reaction was loaded onto the streptavidin column equilibrated with the binding buffer and incubated for another 1 h at room temperature with gentle shaking. Subsequently, the agarose was washed 4–5 times with the binding buffer. After the final wash, the supernatant was aspirated and 10 μL was left above the beads. For protein separation, 20–30 μL pf the SDS loading buffer was added onto the agarose, boiled at 95 °C for 5 min and the sample thus obtained was utilized for electrophoresis.

### Deletion mutagenesis

For deletion mutagenesis, a pair of primers flanking the sequence to be deleted (non-overlapping) was designed. The pCR-Blunt II-TOPO vector (Life Technologies) clone for the *SfUGT33F28* exon 1–2 minigene was utilized as a template. Sequence was successfully amplified using Phusion High Fidelity DNA Polymerase (New England Biolabs) (PCR protocol: 30 s at 98 °C; 20 cycles of 10 s at 98 °C, 30 s at 55–60 °C, 4 min at 72 °C; and 10 min at 72 °C). A *Dpn*I digest was performed to remove the background DNA, followed by ligation and transformation into fresh cells. The sequence of the mutant TOPO clone was then confirmed and utilized as a template for cloning into pIB/V5-His-TOPO vector (Life Technologies) for transfection into insect cells.

### Cloning and heterologous expression of SfUGTs

Sequences were amplified from *S. frugiperda* gut cDNA samples using Phusion High Fidelity DNA Polymerase (New England Biolabs) (PCR protocol: 30 s at 98 °C; 35 cycles of 10 s at 98 °C, 20 s at 55–60 °C, 45 s at 72 °C; and 5 min at 72 °C). The resulting amplified products were purified with a PCR cleanup kit (Qiagen) and incubated with GoTaq DNA polymerase (Promega) for 15 min at 72 °C in order to add A overhangs. The products were cloned into the pIB/V5-His-TOPO vector (Life Technologies) and transformed into NEB cells (Life Technologies), which were plated on selective LB agar medium containing 100 μg/mL ampicillin and incubated overnight at 37 °C. Positive colonies were identified by PCR using vector-specific *OpIE2* primers, sub-cultured overnight at 37 °C in liquid LB medium containing 100 μg/mL ampicillin and used for plasmid DNA purification with the NucleoSpin Plasmid kit (Macherey-Nagel). Concentration and purity of the obtained constructs were assessed by NanoDrop 2000 UV–Vis Spectrophotometer (Thermo Scientific) and the correct orientation of the PCR products was confirmed by DNA sequencing.

### Insect cell transfection

For transfection, Sf9 cells and Hi5 cells were sub-cultured at full confluency in a 6-well plate in a 1:3 dilution and left overnight to adhere to the flask surface. The medium was replaced, and transfections were carried out using FuGENE HD Transfection Reagent (Promega) in a 1:3 plasmid/lipid ratio (1.7 μg plasmid and 5.0 μL lipid for 3 mL medium). Cells were incubated for 48–72 h at 27 °C and re-suspended in fresh medium containing 50 μg/mL blasticidin for 2 weeks. Stable cell cultures were subsequently maintained at 10 μg/mL blasticidin.

### Cell lysate preparation

Cells were obtained from cultures 2 weeks post transfection growing stably on 50 μg/mL blasticidin. A 1 mL quantity of cells was harvested for each construct and re-suspended into 100 µL buffer. Protein concentrations were measured using the Bradford reagent, and 1–2 μg of the cell lysate was used for enzyme assays.

### Microsome preparation

For microsome extraction, confluent, stably transfected cells from five T-75 flasks (10 mL culture) per recombinant plasmid were harvested by scraping the cells off the bottom using a sterile cell scraper (Sarstedt AG, Nuembrecht, Germany). The obtained cell suspensions were combined into a 50 mL falcon tube and centrifuged at 1000×*g* for 15 min at 4 °C (AvantiTM J-20 XP Centrifuge, Beckman Coulter, Krefeld, Germany). The supernatant was discarded, the cells were washed twice with ice-cold PBS buffer (pH 7.4) and centrifuged at 1000×*g* for 15 min. The resulting cell pellet was re-suspended in 10 mL hypotonic buffer (20 mM Tris, 5 mM EDTA, 1 mM DTT, 20% glycerol, pH 7.5), containing 0.1% BenzonaseR nuclease and 100 μL Protease Inhibitor Cocktail (Serva) followed by incubation on ice for 30 min. After cell lysis, the cells were homogenized by 20–30 strokes in a Potter–Elvehjem tissue grinder (Kontes Glass Co., Vineland, USA) and were subsequently mixed with an equal volume of sucrose buffer (20 mM Tris, 5 mM EDTA, 1 mM DTT, 500 mM sucrose, 20% glycerol, pH 7.5). The homogenate was centrifuged at 1200×*g* and 4 °C for 10 min (AvantiTM J-20 XP Centrifuge, Beckman Coulter), and the supernatant was transferred into Beckman polycarbonate ultracentrifugation bottles (25 × 89 mm) (Beckman Coulter) and centrifuged at 100,000×*g* and 4 °C for 1.5 h in a fixed angle Type 70 Ti rotor (OptimaTM L-90K Ultracentrifuge, Beckman Coulter). After ultracentrifugation, the clear supernatant, containing the cytosolic fraction, was aliquoted into 1.5 mL Eppendorf tubes. The pellet, containing the microsomal fractions, was re-suspended in 1 mL of phosphate buffer (100 mM K_2_HPO_4_, pH 7.0), containing 10 μL Protease Inhibitor Cocktail (Serva) and stored at − 80 °C until further use. Typically, 5–10 μg of the microsome fraction so obtained was utilized for the enzyme assays.

### Cross-linking assays

Cross-linking assays were performed using dimethyl suberimidate (DMS) as the cross-linking agent. A fresh stock of DMS (5 mg/mL) was prepared in 0.2 M triethanolamine (pH 8.0) at the start of each assay. DMS was added to a final concentration of 2.5 mg/mL to insect cell microsomes with gentle shaking up to 3 h, and samples were subsequently stored at − 20 °C until further use. All protein samples were electrophoresed using a 12% Mini-PROTEAN tris glycine gel, blotted onto PVDF membrane using wet transfer at 70 V for 30–45 min, followed by detection using the V5-HRP conjugate.

### V5-based affinity purification

Anti-V5 agarose affinity gel (Sigma-Aldrich) was employed for protein purification. Briefly, 50–75 μL of the agarose was packed into a 1.5 mL Eppendorf tube for each sample. The agarose was allowed to settle with a short centrifugation and the supernatant was discarded. The agarose was washed 4–5 times with PBS (pH 7.4). Samples to be purified were incubated with 5% digitonin on ice for 20 min and subject to centrifugation at 16,000×*g* for 30 min. Clarified cell lysate or microsomal extract was added onto the resin (up to 200 μL, volume adjusted by addition of PBS) and incubated for 1.5 h on a shaker. Subsequently, the agarose was washed 4–5 times with PBS. After the final wash, the supernatant was aspirated and 10 μL was left above the beads. This fraction was used for both protein electrophoresis and enzyme assays (separate purifications). For SDS-PAGE, 20–30 μL pf the SDS loading buffer was added onto the agarose, boiled at 95 °C for 5 min and sample thus obtained was utilized for electrophoresis.

### LC–MS/MS peptide analysis

Protein bands of Coomassie Brilliant blue R250 stained gels were cut from the gel matrix and tryptic digestion was carried out^63^. For LC–MS/MS analysis of the resulting peptides, samples were reconstituted in 20 μL aqueous 1% formic acid, and 1 μL was injected onto an UPLC M-class system (Waters, Manchester, UK) coupled to a Synapt G2-si mass spectrometer (Waters, Manchester, UK). Samples were first pre-concentrated and desalted using a Symmetry C18 trap column (100 Å, 180 µm × 20 mm, 5 µm particle size) at a flow rate of 15 µL/min (0.1% aqueous formic acid). Peptides were eluted onto a ACQUITY UPLC HSS T3 analytical column (100 Å, 75 µm × 200 mm, 1.8 µm particle size) at a flow rate of 350 nL/min with the following gradient: 3–15% over 3 min, 15–20% B over 7 min, 20–40% B over 30 min, 40–50% B over 5 min, 50–70% B over 5 min, 70–95% B over 3 min, isocratic at 95% B for 1 min, and a return to 1% B over 1 min. Phases A and B were composed of 0.1% formic acid and 100% acetonitrile in 0.1% formic acid, respectively). The analytical column was re-equilibrated for 10 min prior to the next injection. The eluted peptides were transferred into the mass spectrometer operated in V-mode with a resolving power of at least 20,000 full width at half height FWHM. All analyses were performed in a positive ESI mode. A 100 fmol/μL sample of human Glu-Fibrinopeptide B in 0.1% formic acid/acetonitrile (1:1 v/v) was infused at a flow rate of 1 μL/min through the reference sprayer every 45 s to compensate for mass shifts in MS and MS/MS fragmentation mode. Data were acquired using data-dependent acquisition (DDA). The acquisition cycle for DDA analysis consisted of a survey scan covering the range of *m/z* 400–1800 Da followed by MS/MS fragmentation of the ten most intense precursor ions collected at 0.5 s intervals in the range of 50–2000 m*/z*. Dynamic exclusion was applied to minimize multiple fragmentations for the same precursor ions. MS data were collected using MassLynx v4.1 software (Waters, Manchester, UK).

### Data processing and protein identification

DDA raw data were processed and searched against a sub-database containing common contaminants (human keratins and trypsin) using ProteinLynx Global Server (PLGS) version 2.5.2 (Waters, Manchester, UK). Spectra remaining unmatched by database searching were interpreted de novo to yield peptide sequences and subjected to homology-based searching using the MS BLAST program^64^ installed on a local server. MS BLAST searches were performed against a *Spodoptera frugiperda* database obtained by in silico translation of the S*. frugiperda* transcriptome^37^ and against *arthropoda* database (NCBI). PKL-files of MS/MS spectra were generated and searched against *Spodoptera frugiperda* database combined with NCBI nr (downloaded on May 24, 2020) using MASCOT software version 2.6.2. The following searching parameters were applied: fixed precursor ion mass tolerance of 15 ppm for the survey peptide, fragment ion mass tolerance of 0.1 Da, 1 missed cleavage, fixed carbamidomethylation of cysteines and possible oxidation of methionine.

### Enzymatic assays

For UGT assays, samples from insect cell cultures (transient or stable) were prepared in phosphate buffer (pH 7.0, 100 mM). Typical enzyme reactions included 5–10 µg cell microsomal extracts, 2 μL of 12.5 mM DIMBOA in DMSO (25 nmol), 4 μL of 12.5 mM UDP-glucose in water (50 nmol), and phosphate buffer (pH 7.0, 100 mM) to give an assay volume of 50 μL. Controls containing either boiled enzymatic preparation, or only the protein suspension and buffer were included. After incubation at 30 °C for 60 min, the enzyme reactions were interrupted by adding 50 μL of 1:1 (v:v) methanol/formic acid solution. For enzyme assays involving resin purified microsomal extracts, equal amounts of extracts were employed for resin purification and the enzyme assay (buffer + substrate) was pipetted directly onto the resin. Post incubation, samples were centrifuged, supernatant was collected, and reaction was stopped by addition of methanol/formic acid solution. Assays were centrifuged at 5000*g* for 5 min and the obtained supernatant was collected and analyzed by LC–MS/MS.

### Chromatographic methods

For all analytical chromatography procedures, formic acid (0.05%) in water and acetonitrile were used as mobile phases A and B, respectively, and the column temperature was maintained at 25 °C. Analyses of enzymatic assays and plant samples used a Zorbax Eclipse XDB-C18 column (50 × 4.6 mm, 1.8 μm, Agilent Technologies) with a flow rate of 1.1 mL/min and with the following elution profile: 0–0.5 min, 95% A; 0.5–6 min, 95–67.5% A; 6.02–7 min, 100% B; 7.1–9.5 min, 95% A. LC–MS/MS analyses were performed on an Agilent 1200 HPLC system (Agilent Technologies) coupled to an API 6500 tandem spectrometer (AB Sciex) equipped with a turbospray ion source operating in negative ionization mode. Multiple reaction monitoring (MRM) was used to monitor analyte parent ion to product ion conversion with parameters from the literature for DIMBOA^65^ and DIMBOA-Glc^16^. Analyst (version 1.6.3, Applied Biosystems) software was used for data acquisition and processing.

### Statistical analysis

All statistical analyses were carried out using SigmaPlot 12.0 and R studio (version 3.6.3). Data were tested for homogeneity of variance and normality and were appropriately transformed to meet these criteria where required. The specific statistical method used for each data set is described in the figure legends.

## Supplementary Information

Supplementary Information.

## Data Availability

The proteomics datasets generated in this study are deposited in an open access repository (10.17617/3.WTCPOX). The raw data generated in this study are provided in the Source Data file. All other data are available within the paper and the supplementary information file.
