# Author Correction: Alternative transcript splicing regulates UDP-glucosyltransferase-catalyzed detoxification of DIMBOA in the fall armyworm (*Spodoptera frugiperda*)

**DOI:** 10.1038/s41598-022-17499-z

**Published:** 2022-08-02

**Authors:** Bhawana Israni, Katrin Luck, Samantha C. W. Römhild, Bettina Raguschke, Natalie Wielsch, Yvonne Hupfer, Michael Reichelt, Aleš Svatoš, Jonathan Gershenzon, Daniel Giddings Vassão

**Affiliations:** Max Planck Institute for Chemical Ecology, Jena, Germany

Correction to: *Scientific Reports* 10.1038/s41598-022-14551-w, published online 20 June 2022

The original version of this Article contained an error in Reference 2, which was incorrectly given as:

Kalleshwaraswamy, C*. et al.* First report of the fall armyworm, *Spodoptera frugiperda* (JE Smith) (Lepidoptera: Noctuidae), an alien invasive pest on maize in India (2018).

The correct reference is listed below:

Sharanabasappa, Kalleshwaraswamy, C. M., Asokan, R., Mahadeva Swamy, H. M., Maruthi, M. S., Pavithra, H. B., Hegde, K., Navi, S., Prabhu, S. T. & Goergen, G. 2018. First report of the Fall armyworm, *Spodoptera frugiperda* (J E Smith) (Lepidoptera: Noctuidae), an alien invasive pest on maize in India. *Pest Manage. Hortic. Ecosyst.*, **24**, 23–29 (2018).

The original Article has been corrected.

