# Supplementary material for: Alternative transcript splicing regulates UDP-glucosyltransferase-catalyzed detoxification of DIMBOA in the fall armyworm (Spodoptera frugiperda)
Source: Sci Rep. 2022 Jun 20;12:10343. doi: 10.1038/s41598-022-14551-w (PMC9209448; doi:10.1038/s41598-022-14551-w)
Supplement: Supplementary file 1 — Supplementary Information. [file 41598_2022_14551_MOESM1_ESM.pdf]

SUPPLEMENTARY INFORMATION

Alternative transcript splicing regulates UDP-glucosyltransferase-catalyzed detoxification of DIMBOA in the fall armyworm (*Spodoptera frugiperda*)

Bhawana Israni, Katrin Luck, Samantha C.W. Römhild, Bettina Raguschke, Natalie Wielsch, Yvonne Hupfer, Michael Reichelt, Aleš Svatoš, Jonathan Gershenzon, Daniel Giddings Vassão

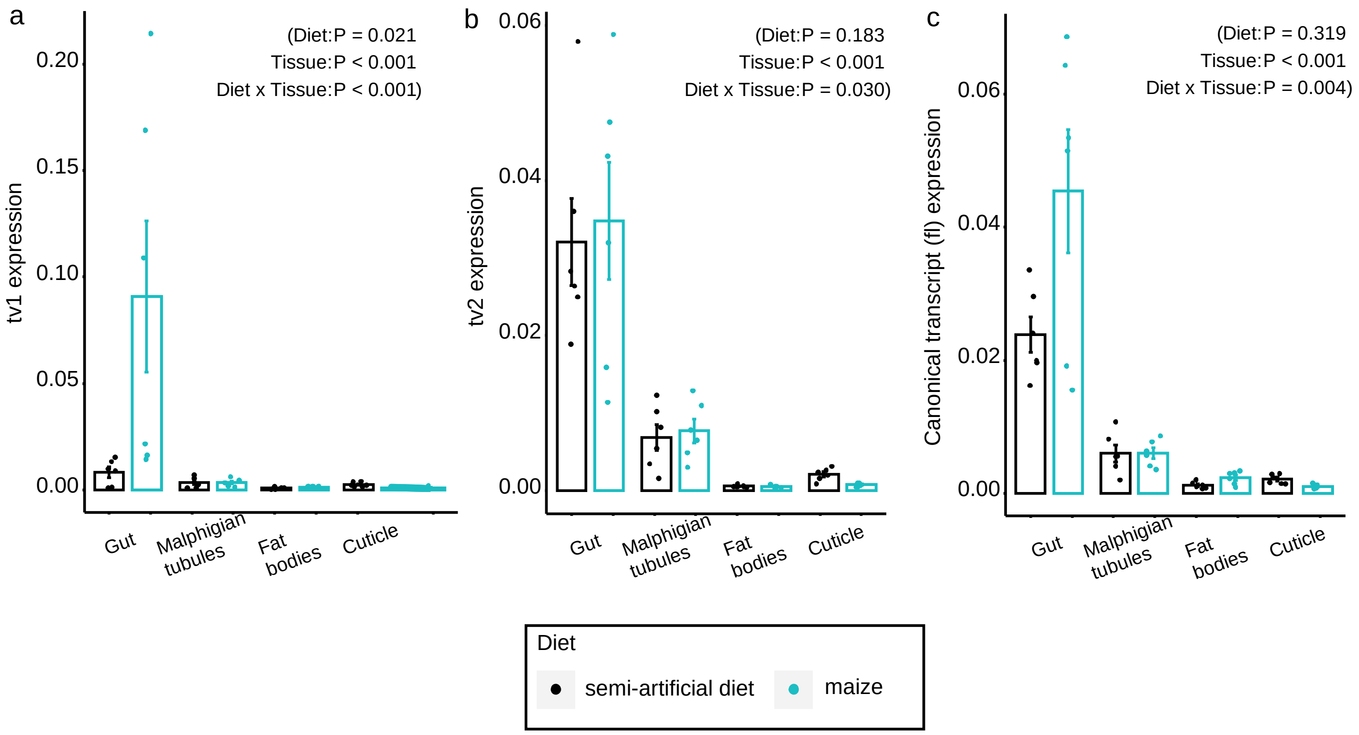

Supplementary figure 1. Real time PCR quantification of expression of *SfUGT33F28* variants in various tissues from caterpillars maintained on semi-artificial (pinto bean-based) diet and maize leaves. Data are presented as mean ± SEM (n=6). All values were log transformed to meet the criteria for normality. Two-way repeated measures ANOVA was performed on log transformed data, and Bonferroni t-test was applied to carry out all pairwise comparisons. tv1, transcript variant 1; tv2, transcript variant 2; fl, full-length.

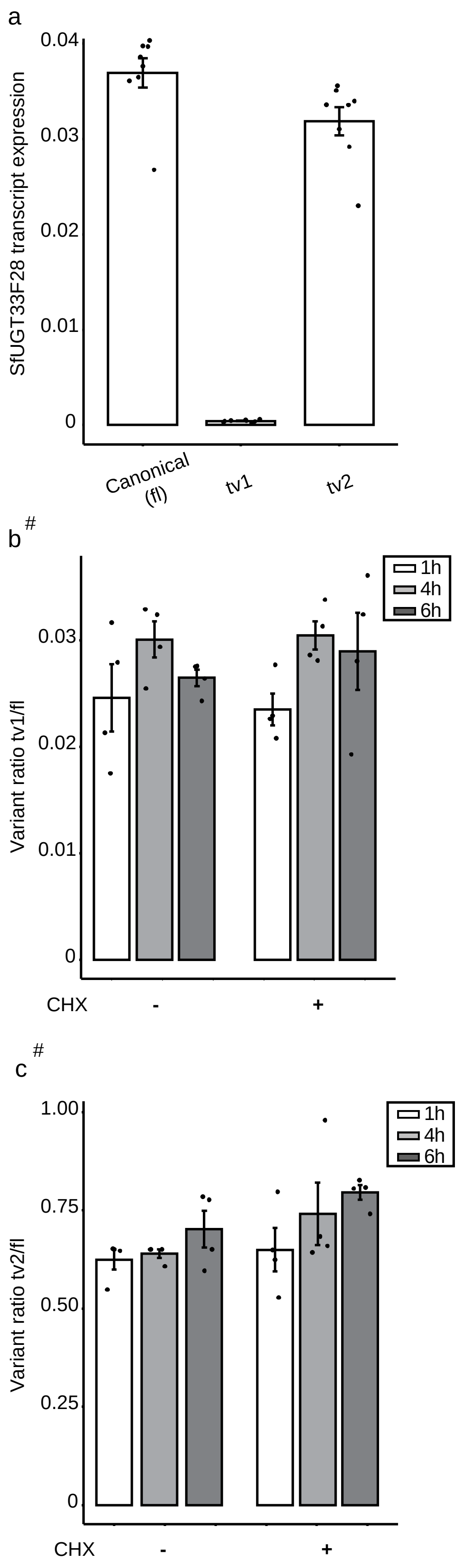
a
SfUGT33F28 transcript expression
0.04
0.03
0.02
0.01
0
Canonical (fl)
tv1
tv2
b #
Variant ratio tv1/fl
0.03
0.02
0.01
0
1h
4h
6h
CHX
-
+
c #
Variant ratio tv2/fl
1.00
0.75
0.50
0.25
0
1h
4h
6h
CHX
-
+

Supplementary figure 2. Expression profile of *SfUGT33F28* transcript variants in insect derived Sf9 cells. a) Quantitation of *SfUGT33F28* transcript levels in untreated Sf9 cells by real time PCR, b) quantitation of *SfUGT33F28* variants tv1 and tv2 with respect to full length canonical transcript upon treatment of Sf9 cells with cycloheximide (CHX) over a period of 1- 6 hours. Data are presented as means ± SEM (n=4-6). One-way ANOVA was performed, and Tukey's test was applied to carry out pairwise comparisons (b-c). # indicates no significant differences between the means. tv1, transcript variant 1; tv2, transcript variant 2; fl, full-length.

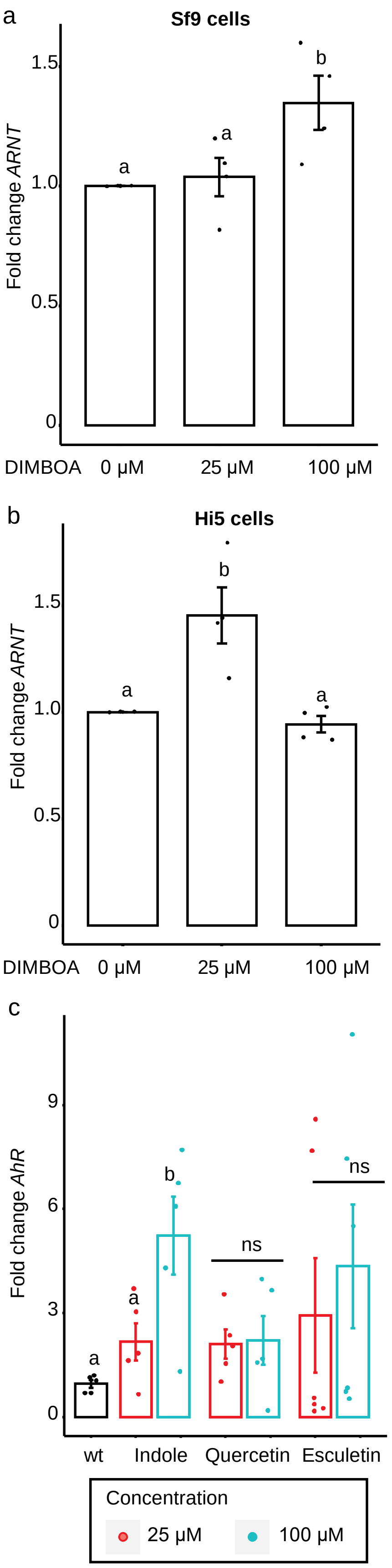

a
Sf9 cells
Fold change ARNT
1.5
1.0
0.5
0
b
a
a
DIMBOA
0 µM
25 µM
100 µM
b
Hi5 cells
Fold change ARNT
1.5
1.0
0.5
0
b
a
a
DIMBOA
0 µM
25 µM
100 µM
c
Fold change AhR
9
6
3
0
b
a
a
ns
ns
wt
Indole
Quercetin
Esculetin
Concentration
25 µM
100 µM

Supplementary figure 3. ***ARNT*** **expression in response to DIMBOA and specificity of** ***AhR*** **responses across a range of chemically diverse substrates. a-b) Quantitation of** ***ARNT*** **transcript levels in untreated and DIMBOA-treated Sf9 and Hi5 insect cells by real time PCR (n=4-5), c) quantitation of** ***AhR*** **transcript levels in insect cells upon treatment with indole, quercetin and esculetin respectively (n=4). All data are presented as mean ± SEM. One-way ANOVA on ranks was performed and Tukey's test was applied to carry out all pairwise comparisons (a, b). One-way ANOVA was performed, and Tukey's test was applied to carry out all pairwise comparisons (c). Small letters on the bars indicate significant differences at P < 0.05. wt, untreated cells.**

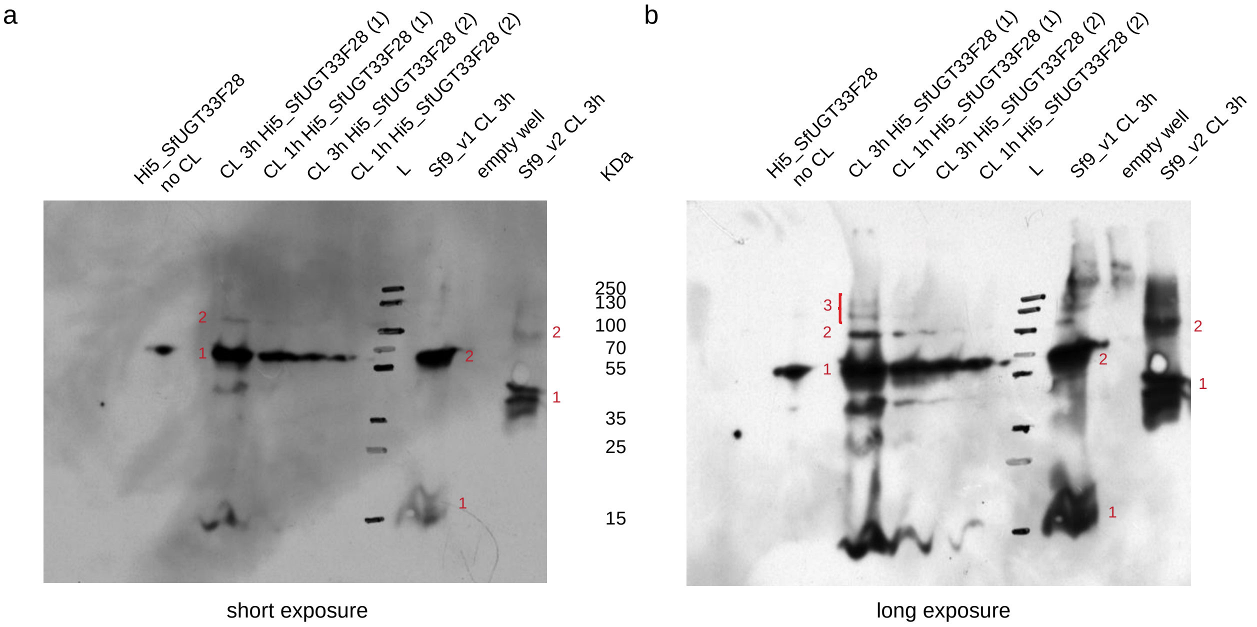

Supplementary figure 4. Cross-linking assays to show the interaction of catalytically inactive UGT variants with other microsomal proteins. Cross-linking assays were performed with Hi5 insect cell microsomes producing UGT33F28 full-length protein, Sf9 insect cell microsomes producing UGT33F28 variants UGT33F28_v1 and UGT33F28_v2 (c) using dimethyl suberimide (DMS). The oligomers observed upon cross-linking are indicated by numbers in red (1: monomer, 2: dimer (homo/hetero), 3: higher order oligomer, L: pre-stained PAGE ruler).

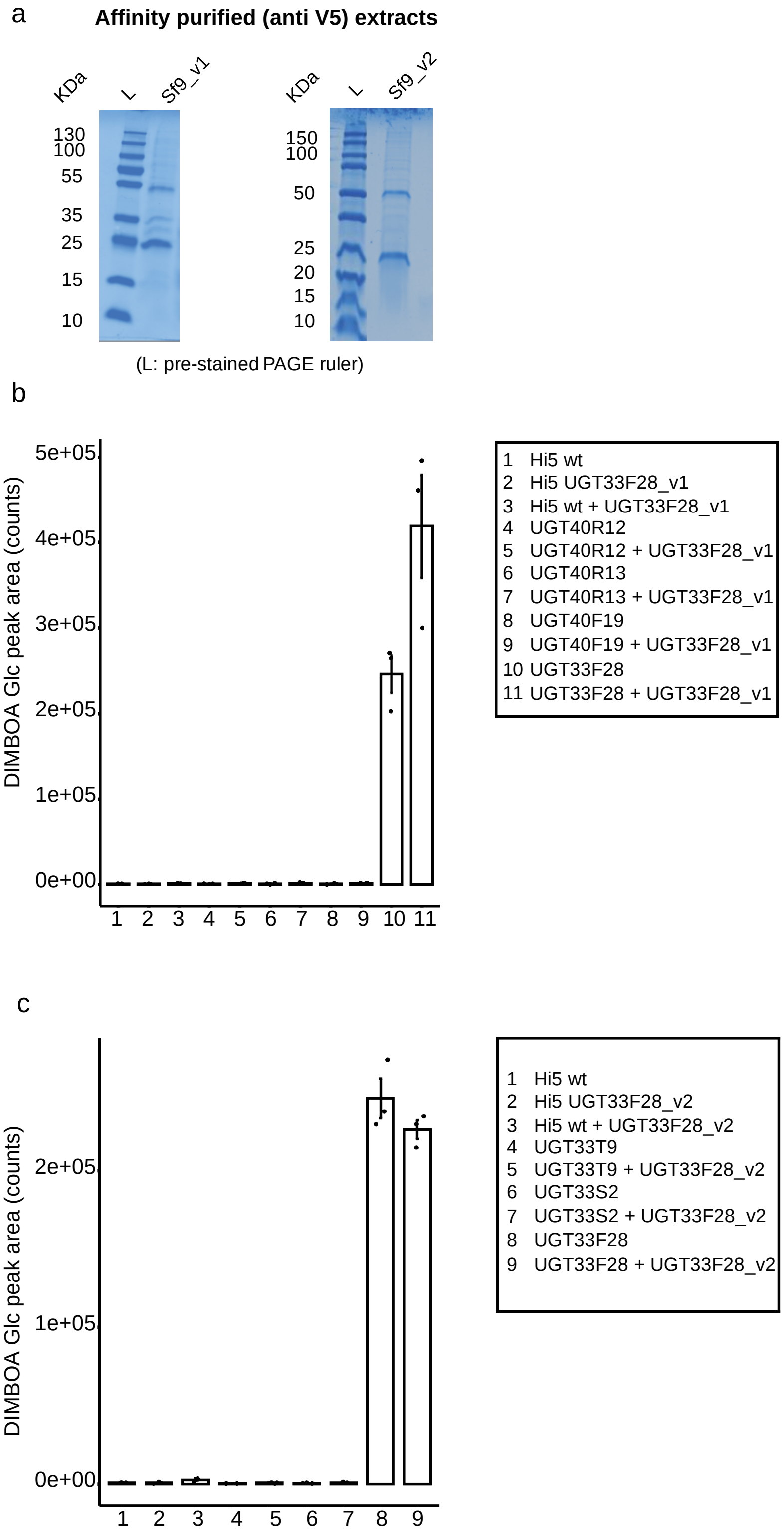

Supplementary figure 5. Interaction and catalytic activities of UGT33F28 variants with full-length UGT33F28 and other proteins predicted from proteomic analyses of bound fractions. a)

Electrophoretic separation of V5 affinity purified fractions from Sf9 cells overexpressing UGT33F28 variants on a 4-20% Mini-PROTEAN tris glycine gel for proteomics and subsequent staining with Coomassie blue, b-c) ***In vitro*** **enzymatic assays were performed using UGTs heterologously produced in Hi5 cells with DIMBOA as substrate. Data are represented as mean ± SEM (n=3).**

**Gene**	**Forward primer (5'-3')**	**Reverse primer (5'-3')**
SfUGT33F28 variant exon 1qPCR	CTAGCTAGAAGAGGCTTACACCAAG	GATCCTGTCAGCTGGTAGTTTAG
SfUGT33F28 canonical exon 1 qPCR	TGTTCCCGAACCCTTCAATC	GACAGTAACTTCATGGCCTCTT
SfUGT33F28 variant exon 3 qPCR	CAAACGTGGACCTGCAGCCA	CGTTTGGCTTTTTTAGGTTTAC
SfUGT33F28 canonical exon 3 qPCR	GTCGTTCAACGCTGAGAGATAC	CGCAGTTTTACCATGTTCTCC
SfRPL10 qPCR	ATTGGACAGCCCATCATGTC	AAGCCCCATTTCTTGGAGAC

Supplementary table 1. Primer pairs used for distinguishing between ***SfUGT33F28*** **mRNA variants.**

**Gene**	**Forward primer (5'-3')**	**Reverse primer (5'-3')**
Exon 1-3 splicing variant	AACTCCCATGAGGGTAAGC	CTCTTATCATTAATAACAGTGTTGATAGC
Exon 1-2 splicing variant	AACTCCCATGAGGGTAAGC	GAGAAGATCAGGCTGTGGC

Supplementary table 2. Primer pairs used for preparation of *SfUGT33F28* minigene.

**Match name/ Accession**	**E- value**	**% Identity**	**Description**
XP_021187219.1	6.700e-279	99.38	aryl hydrocarbon receptor protein 1 [Helicoverpa armigera]
XP_022815375.1	6.700e-279	99.38	aryl hydrocarbon receptor isoform X2 [Spodoptera litura]
PCG63465.1	1.900e-278	100.00	hypothetical protein B5V51_12264, partial [Heliothis virescens]
XP_026735439.1	2.500e-278	99.17	aryl hydrocarbon receptor [Trichoplusia ni]
XP_026761162.1	1.300e-271	96.68	aryl hydrocarbon receptor [Galleria mellonella]
XP_028157966.1	3.700e-269	96.69	aryl hydrocarbon receptor isoform X1 [Ostrinia furnacalis]
XP_026323827.1	1.100e-268	95.44	aryl hydrocarbon receptor isoform X2 [Hyposmocoma kahamanoa]
XP_022815374.1	5.300e-268	99.35	aryl hydrocarbon receptor isoform X1 [Spodoptera litura]
XP_013165847.1	6.100e-264	94.19	PREDICTED: aryl hydrocarbon receptor, partial [Papilio xuthus]
KPJ03486.1	6.100e-264	94.19	Aryl hydrocarbon receptor [Papilio xuthus]

Supplementary table 3. Description of top scoring BLAST hits using putative SfAhR protein sequence as a query against GenBank protein database.

**Match name/ Accession**	**E- value**	**% Identity**	**Description**

XP_022837764.1	6.000e-291	98.63	aryl hydrocarbon receptor nuclear translocator-like protein 2 [Spodoptera litura]
XP_021188262.1	2.100e-280	94.72	aryl hydrocarbon receptor nuclear translocator-like protein 2 [Helicoverpa armigera]
XP_026734341.1	1.500e-273	92.37	aryl hydrocarbon receptor nuclear translocator-like protein 1 [Trichoplusia ni]
AZL94115.1	2.500e-273	92.56	juvenile hormone methoprene tolerant protein [Mythimna separata]
XP_026492150.1	8.800e-226	75.98	aryl hydrocarbon receptor nuclear translocator-like protein 1 [Vanessa tameamea]
KOB74415.1	1.300e-221	77.12	Methoprene tolerant protein 1 [Operophtera brumata]
ANZ54967.1	3.200e-220	77.17	juvenile hormone receptor methoprene-tolerant [Plodia interpunctella]
XP_013186860.1	5.500e-220	76.28	PREDICTED: aryl hydrocarbon receptor nuclear translocator-like protein 1 [Amyelois transitella]
XP_028173519.1	3.600e-219	75.10	aryl hydrocarbon receptor nuclear translocator-like protein 2 [Ostrinia furnacalis]
XP_026761772.1	1.400e-215	73.62	aryl hydrocarbon receptor nuclear translocator-like

			protein 1 [Galleria mellonella]

Supplementary table 4. Description of top scoring BLAST hits using putative SfARNT protein sequence as a query against GenBank_protein database.

**Organism**	**Description**	**Predicted NLS**
Homo sapiens (NP_001659)	Aryl hydrocarbon receptor nuclear translocator isoform 1	RAIKRRPGLD (monopartite)
Drosophila melanogaster (AAB69695)	Aryl hydrocarbon receptor nuclear translocator-like protein	-
Spodoptera frugiperda (GSSPFG00020774001)	Aryl hydrocarbon receptor nuclear translocator	RFDSRKRRKVD, DSRKRRKVDC (monopartite)
Trichoplusia ni (XP_026734341.1)	Aryl hydrocarbon receptor nuclear translocator-like protein 1	RFDSRKRRKID, DSRKRRKIDC (monopartite)

Supplementary table 5. ARNT proteins from select organisms with their predicted nuclear localization signal (NLS). Prediction of NLS in ARNT proteins from *H. sapiens, D. melanogaster, S. frugiperda* and *T.ni* was carried using the cNLS mapper. NLS prediction was carried out using a cut-off score of 5.

**Gene**	**Forward primer (5’-3’)**	**Reverse primer (5’-3’)**
SfAhR/ *TnAhR* qPCR	GCTTACCGCTTCCAAACAAAG	GCGGTCGATGCGTACTAATA
SfARNT/ *TnARNT* qPCR (1)	GTGTTATTGGAGAAGATTATGGA	CATCATTGGCTGCTTGTT
SfARNT/ *TnARNT* qPCR (2)	GTATGCCTACCGCAAAAGCAAG	GCACTATATCCGACAACAACGAC

Supplementary table 6. Real time primer pairs used for estimation of *AhR* and *ARNT* transcript levels in insect cells.

Gene	Forward primer (5'-3')	Reverse primer (5'-3')
Canonical DNA probe 1 (wt) *SfUGT33F28*	TACGCGTGTTATTGAACTTC	GAAGTTCAATAACACGCGTA
Mutant DNA probe 2 (Δahr/arnt) *SfUGT33F28*	TATTATTGAACTTCAAAACATAC	GTATGTTTTGAAGTTCAATAATA
Unlabeled canonical DNA probe 1 (wt) *SfUGT33F28*	TACGCGTGTTATTGAACTTC	GAAGTTCAATAACACGCGTA
SfUGT33F28 : Δahr/arnt	AATAGATATTATTGAACTTCAAAACATACGG	TCAATAATATCTATTTGTAGTTAAATGTTAGA

Supplementary table 7. Probes used for EMSA for detection of AhR-ARNT binding to *SfUGT33F28* gene, and subsequent deletion mutagenesis in *SfUGT33F28* 5' UTR.

Accession	Mascot DB	Mascot Description	Mascot Good Peptides	Mascot Score
GSSPFG00013717001	Spodoptera_Frugiperda_PRO_20151204_X	Putative aryl hydrocarbon receptor	1	57
GSSPFG00006764001	Spodoptera_Frugiperda_PRO_20151204_X	EH domain-containing protein isoform	4	267

GSSPFG00005966001.4	Spodoptera_Frugiperda_PRO_20151204_X	kinetochore protein NDC80 homolog	4	236
GSSPFG00019338001.3	Spodoptera_Frugiperda_PRO_20151204_X	Heat shock protein 70-2	3	182
GSSPFG00011292001.1	Spodoptera_Frugiperda_PRO_20151204_X	ATP-dependent RNA helicase WM6	2	170
GSSPFG00003602001.1	Spodoptera_Frugiperda_PRO_20151204_X	Protein disulfide isomerase	2	141
GSSPFG00018584001	Spodoptera_Frugiperda_PRO_20151204_X	putative ATP-dependent RNA helicase DDX3X	2	137
GSSPFG00034579001.2	Spodoptera_Frugiperda_PRO_20151204_X	Eukaryotic initiation factor 2%CE%B3	2	137
GSSPFG00025750001.2	Spodoptera_Frugiperda_PRO_20151204_X	gene=putative pre-mRNA-splicing factor ATP-dependent RNA helicase DHX15-like	2	128
GSSPFG00034579001.2	Spodoptera_Frugiperda_PRO_20151204_X	initiation factor	2	127
GSSPFG00025091001.1	Spodoptera_Frugiperda_PRO_20151204_X	26S regulatory subunit	3	124
GSSPFG00010067001	Spodoptera_Frugiperda_PRO_20151204_X	histone deacetylase Rpd3 partial [Anopheles gambiae]	3	117
GSSPFG00010630001.2	Spodoptera_Frugiperda_PRO_20151204_X	putative loquacious	1	89
GSSPFG00008503001	Spodoptera_Frugiperda_PRO_20151204_X	cyclin-K-like gene=GSSPFG00008503001 [Spodoptera litura]	1	72
GSSPFG00035947001.2	Spodoptera_Frugiperda_PRO_20151204_X	Suppressor of variegation 3-9	1	70
GSSPFG00027194001	Spodoptera_Frugiperda_PRO_20151204_X	KH domain-containing RNA-binding signal transduction-associated protein 2-like isoform [Helicoverpa armigera] X1 [Spodoptera litura]	1	70

GSSPFG00012808001.3	Spodoptera_Frugiperda_PRO_20151204_X	26S proteasome regulatory ATPase subunit 10b	1	68
GSSPFG00005183001.3	Spodoptera_Frugiperda_PRO_20151204_X	gene=UDP-glycosyltransferase-40-05 gene=UDP-glycosyltransferase-40-11	1	61
XP_021172547.1	NCBIprot_20200524	period circadian protein homolog 2-like [Fundulus heteroclitus]	5	397
XP_022815275.1	NCBIprot_20200524	EH domain-containing protein 3 [Spodoptera litura]	4	267
AAF54856.2	NCBIprot_20200524	Putative achaete scute target 1, isoform B [Drosophila melanogaster]	1	73

Supplementary table 8. Overview of protein hits predicted from the insect cell- nuclear protein fraction bound with biotinylated *SfUGT33F28* DNA probe using streptavidin-based affinity purification.

**Accession**	**Mascot DB**	**Mascot Description**	**Mascot Good Peptides**	**Mascot Score**
GSSPFG00018237001.3	Spodoptera_Frugiperda_PRO_20151204_X	UDP-glycosyltransferase-33-13	3	206
GSSPFG00005183001.3	Spodoptera_Frugiperda_PRO_20151204_X	UDP-glycosyltransferase-40-05	2	69
GSSPFG00008268001.2	Spodoptera_Frugiperda_PRO_20151204_X	CYP9A30	3	192
GSSPFG00004352001	Spodoptera_Frugiperda_PRO_20151204_X	UDP-glycosyltransferase-40-11	2	151
GSSPFG00015767001	Spodoptera_Frugiperda_PRO_20151204_X	Heat shock protein 60	5	319

GSSPFG00034655001	Spodoptera_Frugiperda_PRO_20151204_X	Protein disulfide isomerase	10	893
GSSPFG00003602001	Spodoptera_Frugiperda_PRO_20151204_X	Protein disulfide isomerase	10	893
GSSPFG00022327001	Spodoptera_Frugiperda_PRO_20151204_X	Niemann-Pick C1 protein isoform [Spodoptera litura]	7	243
GSSPFG00015253001	Spodoptera_Frugiperda_PRO_20151204_X	probable citrate synthase mitochondrial	14	712
GSSPFG00026106001.1	Spodoptera_Frugiperda_PRO_20151204_X	isocitrate dehydrogenase [NADP] cytoplasmic-like [Spodoptera litura]	5	249
GSSPFG00001134001.1	Spodoptera_Frugiperda_PRO_20151204_X	cytochrome uncharacterized oxidase protein subunit	3	183
GSSPFG00007626001	Spodoptera_Frugiperda_PRO_20151204_X	ATP PROTEIN synthase subunit	3	171
GSSPFG00031969001.5	Spodoptera_Frugiperda_PRO_20151204_X	molecular heat chaperone shock DnaK protein	8	458
GSSPFG00008691001.3	Spodoptera_Frugiperda_PRO_20151204_X	voltage-dependent anion-selective channel	2	157
GSSPFG00024222001.2	Spodoptera_Frugiperda_PRO_20151204_X	UGT41-03	2	181

Supplementary table 9. Overview of protein hits predicted from V5 affinity purified fractions of Sf9 cells overexpressing SfUGT33F28_v1.

Accession	Mascot DB	Mascot Description	Mascot Good Peptides	Mascot Score
GSSPFG00018237001.3	Spodoptera_Frugiperda_PRO_20151204_X	UDP-glycosyltransferase-33-13	1	58
GSSPFG00014039001	Spodoptera_Frugiperda_PRO_20151204_X	CYP314A1	1	58
GSSPFG00010087001	Spodoptera_Frugiperda_PRO_20151204_X	carboxylesterase 021c	1	61
GSSPFG00013717001	Spodoptera_Frugiperda_PRO_20151204_X	Putative aryl hydrocarbon receptor	1	57
GSSPFG00009831001.2	Spodoptera_Frugiperda_PRO_20151204_X	CYP6AN4	1	57
GSSPFG00026800001.3	Spodoptera_Frugiperda_PRO_20151204_X	CYP9A27PARTIAL	1	56
GSSPFG00000365001.3	Spodoptera_Frugiperda_PRO_20151204_X	Serine protease	2	110
GSSPFG00015767001.3	Spodoptera_Frugiperda_PRO_20151204_X	Heat shock protein 60	1	63
GSSPFG00001834001.5	Spodoptera_Frugiperda_PRO_20151204_X	carboxylesterase 016d	1	58

Supplementary table 10. Overview of protein hits predicted from V5 affinity purified fractions of Sf9 cells overexpressing SfUGT33F28_v2.

Gene	Accession	Forward primer (5'-3')	Reverse primer (5'-3')
SfUGT33F28	GSSPFG00018237001.3	ACCATGAAGATATTAATTTGTATAACTCTG	CGTTTGGCTTTTTTAGGTTTAC
SfUGT33F28 tv 1	GSSPFG00018237001.3	ACCATGAAGATATTAATTTGTATAACTCTG	GGTCACAGTACCAGTCACAGT
SfUGT33F28 tv 2	GSSPFG00018237001.3	ACCATGAAGATATTAATTTGTATAACTCTG	GTTCGAGGTACTCTGCCCA

SfUGT40 R12	GSSPFG00 004145001	GTGCCGATAATGGCCTTAGT ATTA	ATTCCTTTTTTTACTATTT ACAATTTTCTT
SfUGT40 R13	GSSPFG00 005183001	TTGTGCGTGCCAATAATGGCG	ATTCTTCTTATCCTTTTTC TTACTATTTAC
SfUGT40 F19	GSSPFG00 005185001	TTAATAATAATGGAAAAGTT AATATGTTTT	ATTCTTCTTCTCTTTTATA TTCTTCTGA
SfUGT33 T9	GSSPFG00 025012001	TCGATCACAATGTCTGTGC	ACTCCGTTTAATCTTAAC ATCACTAA
SfUGT33 S2	GSSPFG00 016675001	AGTGAGAACATGTTGCTGTG C	GTCTGCCTTAAGCTTCTTT AAAGT

Supplementary table 11. Primer pairs used for cloning and heterologous expression of SfUGTs.

Original blots and gels

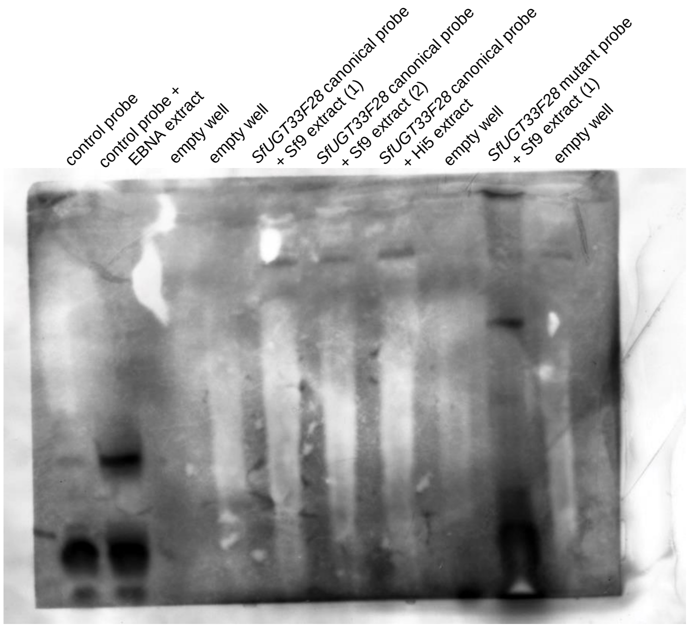

Electrophoretic mobility shift assay (EMSA) to demonstrate the binding of the AhR-ARNT protein complex from insect cells to the nucleotide sequence derived from *SfUGT33F28* 5’ UTR.

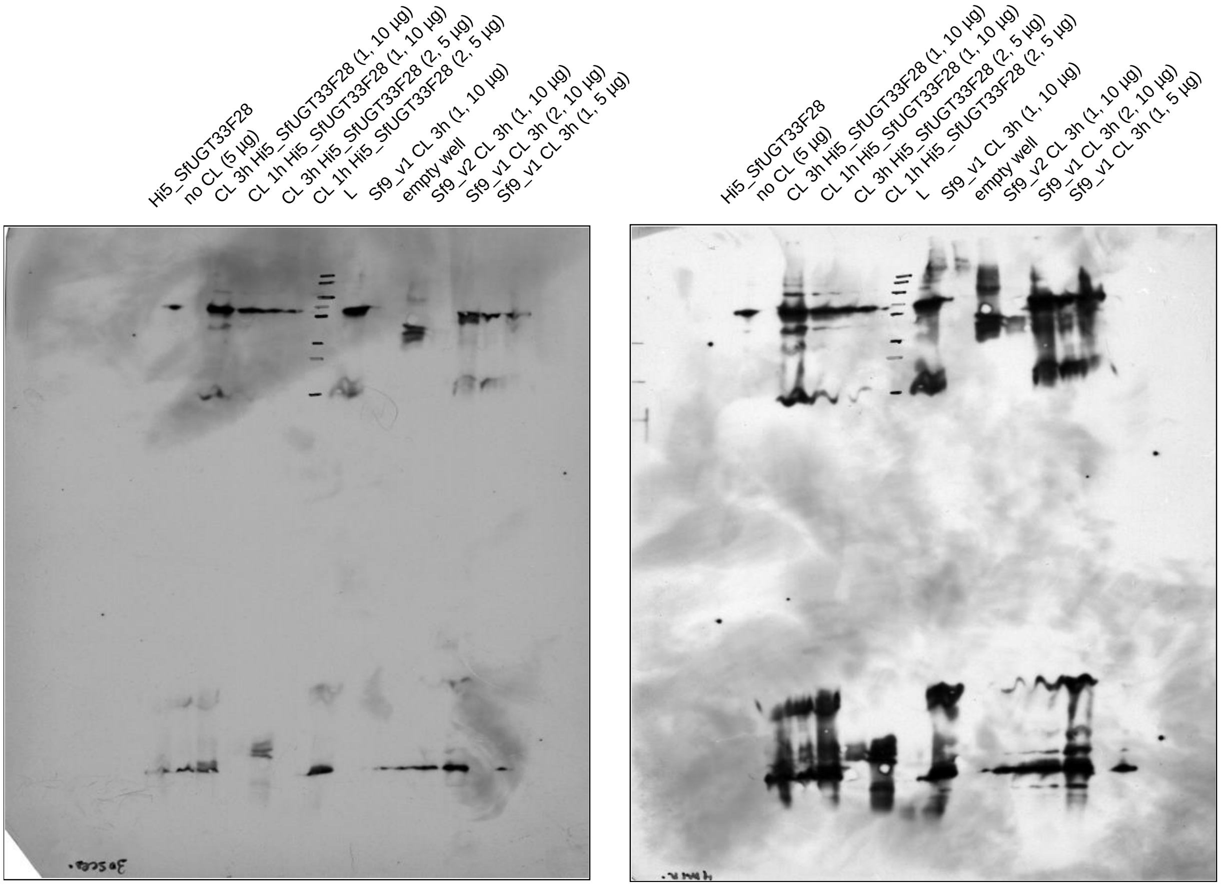

Cross-linking assays to show the interaction of catalytically inactive UGT variants with other microsomal proteins. Cross-linking assays were performed with Hi5 insect cell microsomes producing UGT33F28 full-length protein, Sf9 insect cell microsomes producing UGT33F28 variants UGT33F28_v1 and UGT33F28_v2 (c) using dimethyl suberimide (DMS). L: pre-stained PAGE ruler.

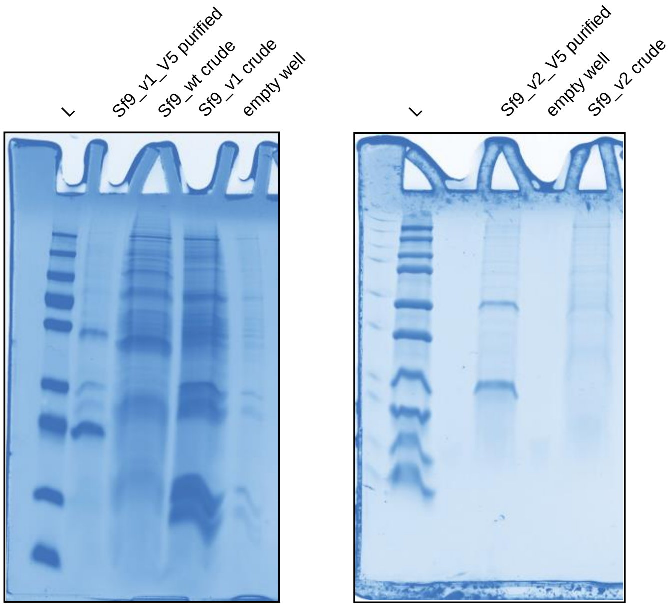

Electrophoretic separation of V5 affinity purified fractions from Sf9 cells overexpressing UGT33F28 variants on a 4-20% Mini-PROTEAN tris glycine gel for proteomics and subsequent staining with Coomassie blue. L: pre-stained PAGE ruler.
